# Spinon mediation of witness spin dynamics in herbertsmithite

**DOI:** 10.1038/s41567-026-03303-6

**Published:** 2026-06-10

**Authors:** Hiroto Takahashi, Jack Murphy, Mitikorn Wood-Thanan, Pascal Puphal, Miguel-Ángel Sánchez-Martínez, Fabian Jerzembeck, Chun-Chih Hsu, Jonathan Ward, Masahiko Isobe, Yosuke Matsumoto, Hidenori Takagi, Stephen J. Blundell, Michael R. Norman, Felix Flicker, J. C. Séamus Davis

**Affiliations:** 1 Clarendon Laboratory, University of Oxford, Oxford, UK; 2 Department of Physics, University College Cork, Cork, Ireland; 3 School of Physics and Astronomy, Cardiff University, Cardiff, UK; 4 School of Physics, University of Bristol, Bristol, UK; 5 Max Planck Institute for Solid State Research, Stuttgart, Germany; 6 Max Planck Institute for Chemical Physics of Solids, Dresden, Germany; 7 Materials Science Division, Argonne National Laboratory, Lemont, IL USA; 8 Department of Physics, Cornell University, Ithaca, NY USA

**Keywords:** Magnetic properties and materials, Quantum fluids and solids

## Abstract

The kagome lattice of spin-1/2 copper atoms in herbertsmithite is conjectured to sustain a quantum spin liquid state with spinon quasiparticles. Ideally, the kagome crystal planes are each separated by a plane of spinless zinc atoms. However, in real crystals, some spin-1/2 copper atoms substitute randomly onto these inter-kagome zinc sites. Here we reconceptualize such ‘impurity’ atoms as quantum witness spins whose dynamics is designed to probe the spin liquid state. We then introduce spin noise spectroscopy to measure the frequency and temperature dependence of witness spin dynamics, demonstrating that their phenomenology is consistent with extensive interactions between witness spins mediated by propagation of spinons through a quantum spin liquid. Ultimately, a sharp transition occurs at around 260 mK, below which the properties of both spin noise and magnetic susceptibility suggest that the witness spins form a spin glass phase. Among the theoretical models considered, we demonstrate that our observations are only consistent with spinon-mediated interactions between witness spins by either a Z_2_ or U(1) quantum spin liquid, with the former model more closely matching the data. Our work demonstrates that quantum mechanical witness spins may now conceivably be used as a widely applicable probe of quantum spin liquid physics.

## Abstract (web summary)

There is a long-running effort to find a material with a spin liquid ground state, but existing experimental probes have not given definitive evidence. Now, impurity spins have been used as probes of the putative spin liquid phase of herbertsmithite.

## Main

In quantum materials research, impurity atoms are usually viewed as pernicious. Yet this can sometimes be misguided. For example, zinc (Zn) impurity atoms substituted at the copper (Cu) site in cuprate high-temperature superconductors provide a prolific source of fundamental understanding^1^. Here we deploy impurity atoms as a resource for the direct atomic-scale study of a putative quantum spin liquid (QSL). In the theory of such materials, strongly interacting spins do not undergo spontaneous magnetic ordering, but enter a ground state with massive long-range quantum entanglement whose fractionalized charge-neutral quasiparticles are dubbed spinons^2–5^. Herbertsmithite, a kagome-lattice magnetic insulator with chemical structure ZnCu_3_(OH)_6_Cl_2_, is a leading candidate to sustain such a QSL state^2–4,6–8^. Moreover, a theoretical analysis of the kagome Heisenberg spin-1/2 Hamiltonian^9,10^ finds that such a QSL exhibits either a spectrum of delocalized spinons above a finite energy gap Δ or of a gapless nature, governed by Z_2_ or U(1) gauge symmetry, respectively. In herbertsmithite, because some spin-1/2 Cu atoms substitute randomly onto the inter-kagome Zn sites^7,8,11^, our objective is to reconceptualize these impurity atoms as ‘witness spins’ to provide an exceptional probe of the conjectured QSL state.

**Fig. 1:**
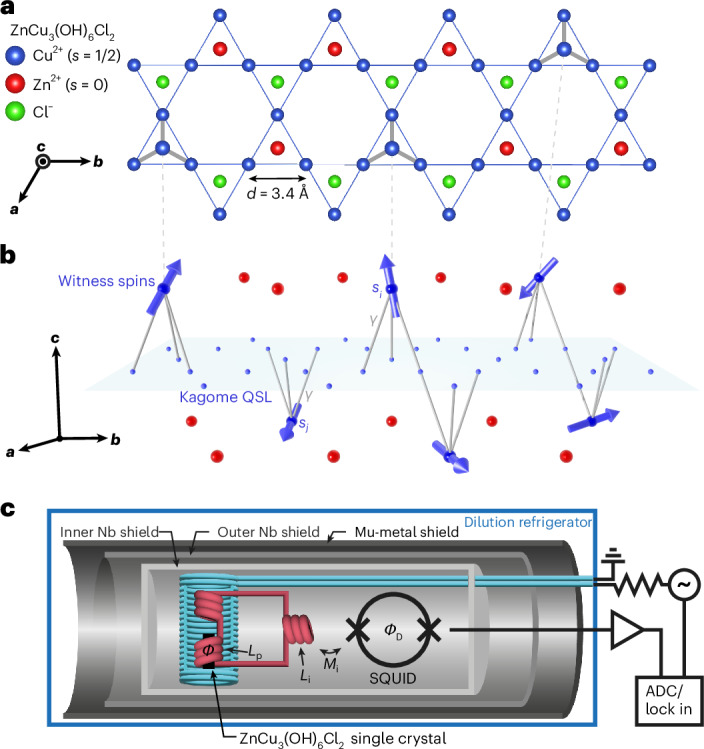
Witness spins in ZnCu_3_(OH)_6_Cl_2_. **a**, Crystal structure of ZnCu_3_(OH)_6_Cl_2_ viewed along the *c* axis showing the kagome plane of Cu^2+^ s=1/2 ions hypothesized to sustain a QSL, and indicating sites of Cu^2+^ s=1/2 ions substituted onto non-magnetic Zn^2+^ s=0 sites. **b**, Interplanar perspective of ZnCu_3_(OH)_6_Cl_2_ again indicating sites of Cu^2+^ s=1/2 ions acting as witness spins substituted on 33% of Zn^2+^ sites. The coupling of each witness spin to three Cu^2+^ spins in the adjacent kagome plane is indicated by pyramidal links. **c**, Schematic of the design of the SQUID spin noise spectrometer that measures the time evolution of flux $\varPhi \left(t\right)$ generated by the ZnCu_3_(OH)_6_Cl_2_ sample in the superconductive pickup coil connected persistently to the SQUID input coil. ADC, analogue-to-digital converter.

The herbertsmithite crystal structure (lattice parameters $a=b=6.84\,\mathring{\rm A}$ and $c=14.09\,{{\mathring{\rm A} }}$) reveals the kagome plane of Cu^2+^ s=1/2 atoms that are hypothesized to sustain a QSL (Fig. 1a). The contribution of these spins alone to the kagome-specific magnetic susceptibility, ${\chi }_{{\rm{K}}}(\omega ,T)$, exhibits a strong downturn at $T\lesssim 30\,{\rm{K}}$, hypothetically due to spin-singlet formation^12,13^. At higher temperatures, the d.c. susceptibility χ(T) indicates an antiferromagnetic (AF) interaction scale ${J}_{{\rm{K}}}\approx 17\,{\rm{meV}}$ between kagome spins, with a congruent Curie–Weiss temperature (${\theta }_{{\rm{CW}}}\approx -300\,{\rm{K}}$) yet no magnetic ordering has been reported down to $T\lesssim 50\,{\rm{mK}}$ (refs. ^14,15^). As the leading QSL candidate, this material has been an intense focus of study, especially on whether the putative spinon spectrum is gapped or gapless. Some nuclear magnetic resonance studies report a spin liquid ground state with a finite energy gap^16^; the low-energy inelastic neutron scattering spectra are reported consistent with a small energy gap in the kagome planes^17^; and pressure tuning of the material reveals an enhancement of the magnetic ordering temperature consistent with a gapped QSL state^18^. On the other hand, there also are nuclear magnetic resonance studies reporting that the kagome plane susceptibility at the lowest temperatures reaches a non-zero value, indicating the absence of an energy gap^13^; both specific heat and susceptibility studies report evidence that herbertsmithite is a QSL without a spin gap^19^; Raman scattering experiments provide further indications of a gapless QSL state^20^; as do optical conductivity experiments^21^ and specific heat measurements^22^. More recent nuclear magnetic resonance studies affirm strongly that the spin excitations in the kagome plane of herbertsmithite are gapless^23^.

There is, however, a second major reservoir of spins outside the kagome planes of herbertsmithite because the substitution of Cu^2+^ s=1/2 ions occurs on approximately 33% of the Zn^2+^ s=0 sites^24^ (see the ‘Herbertsmithite samples’ section). From this interplanar perspective (Fig. 1b), each kagome crystal plane separates two planes of Zn^2+^ atoms (red) into which these Cu^2+^ atoms (blue) substitute. Due to microscopically unexplained AF interactions between these Cu^2+^/Zn^2+^-substituted spins^14^, the residual susceptibility as T→0 evolves approximately as ${\chi }^{-1}\left(T\right)\propto (T-{\theta }_{{\rm{CW}}})$ with an extremely low Curie–Weiss temperature ${\theta }_{{\rm{CW}}}\approx -1.1\,{\rm{K}}$. Equivalently, in $T{\to} 0$ neutron scattering studies, the higher-energy structure factor ${\Sigma} ({\boldsymbol{q}},\omega )$ exhibits a broad continuum^17,25,26^ possibly associated with spinons but, for ${\hslash} {\omega} {\lesssim} 1\,{\rm{meV}}$, ${\Sigma} ({\boldsymbol{q}},\omega )$ is fairly distinct and has been interpreted as due to AF interactions between Cu^2+^/Zn^2+^-substituted spins, driven by interactions thought to be transmitted through the kagome plane^17,27^. A major challenge to determine whether the kagome plane spin excitation spectrum is gapped^16^ or gapless^23^ has been to accurately quantify and physically understand the spin dynamics phenomenology of these Cu^2+^/Zn^2+^-substituted spins.

**Extended Data Fig. 1:**
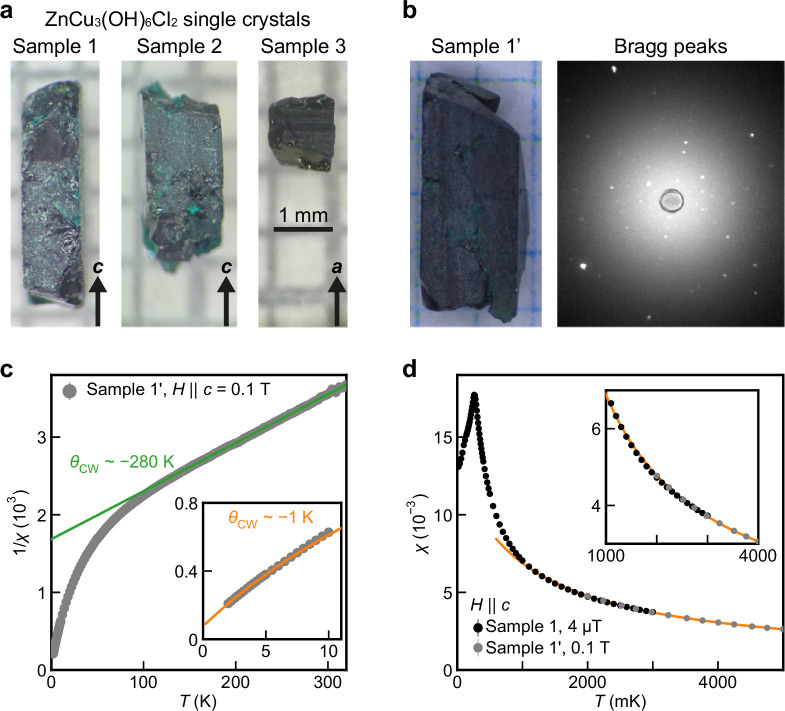
Single crystals of ZnCu_3_(OH)_6_Cl_2_ and their characterization. **a**. Photos of three ZnCu_3_(OH)_6_Cl_2_ single crystals. Sample 1 is obtained by polishing down Sample 1’ in **b**. Measurements were performed along the *c* axis for Samples 1 and 2, and along the *a* axis for Sample 3, as indicated by the arrows. **b**. Clear Bragg peaks of the X-ray Laue diffraction measured for Sample 1’. **c**. DC susceptibility of Sample 1’ in SI units measured by a SQUID MPMS (Quantum Design). The solid lines represent Curie-Weiss fittings by $\chi =\,{\chi }_{0}+\frac{{C}_{{\rm{Curie}}}}{T-{\theta }_{{\rm{CW}}}}$. Fitting in a temperature range 150 K $\le T\le$ 320 K (green) yields ${\chi }_{0}=-5\times {10}^{-6}$, ${\theta }_{{\rm{CW}}}=-280$ K, and ${C}_{{\rm{Curie}}}=0.165$ K and that in a temperature range 2 K $\le T\le$ 6 K (orange, in inset) yields ${\chi }_{0}=4.3\times {10}^{-4}$, ${\theta }_{{\rm{CW}}}=-1.07$ K, and ${C}_{{\rm{Curie}}}=0.0134$ K. **d**. The overlay of the micro-Tesla susceptibility in Fig. 3c (black) and sub-Tesla susceptibility in **c** (gray). The inset magnifies the overlapping temperature range. The fitting in the range 2 K $\le T\le$ 6 K (orange) captures the susceptibility down to 1 K. Source data

Although such substitutional impurity spins are widely viewed as an impediment to analysis of the T→0 phenomenology of herbertsmithite^7,8^ (Supplementary Discussion), here we exploit them as an innovative resource. Viewed as quantum mechanical witness spins, they may conceivably be used for exploration of the QSL state itself ^23,28^, through its influence on their spin dynamics. To explore this concept, we introduce spin noise spectroscopy^29–33^ to QSL studies, using a custom-built spin noise spectrometer (Fig. 1c) measuring the time evolution of flux Φ(t) generated by ZnCu_3_(OH)_6_Cl_2_ crystals. The apparatus uses a pair of opposite-chirality superconducting solenoids (Fig. 1c) in a continuous superconductive circuit including the input inductance to a d.c. superconducting quantum interference device (SQUID). The output voltage of the SQUID is ${V}_{{\rm{D}}}\left(t\right)\equiv {\varPhi }_{{\rm{D}}}\left(t\right) / \eta$, where ${\varPhi }_{{\rm{D}}}(t)$ is the flux delivered to the SQUID and $\eta =0.10\ {\varPhi }_{0} / {\rm{V}}$; the measured flux ${\varPhi }_{{\rm{D}}}(t)$ relates to the flux threading the sample \varPhi (t\right) as ${\varPhi }_{{\rm{D}}}(t)\equiv \beta \varPhi (t)$, where for this apparatus, β=0.0084. Our spin noise spectrometer achieves magnetic field sensitivity ${\mu }_{0}\delta {M}\le {10}^{-14}\ {\rm{T}} / \sqrt{{\rm{Hz}}}$, on a cryogen-free ultralow-vibration dilution refrigerator in the range of $10\,\mathrm{mK}\le T\le 5,000\,\mathrm{mK}$. The time sequence of the magnetic flux Φ(t) generated by the sample magnetization ${M}(t)=\vartheta \varPhi (t)/{\mu }_{0}$ within the pickup coil, where $\vartheta =1.1\times {10}^{-10}\ \rm{T}/{\varPhi }_{0}$, can be measured with microsecond precision. In a given experiment, typical measurables include ${M}(t,T)$, ${S}_{{M}}(\omega ,T)$, ${\sigma }_{{M}}^{2}(T)$ and χ(T) (refs. ^29–33^). Our preparation and evaluation procedure for the ZnCu_3_(OH)_6_Cl_2_ sample (Extended Data Fig. 1) are described in the ‘Herbertsmithite samples’ section.

**Fig. 2:**
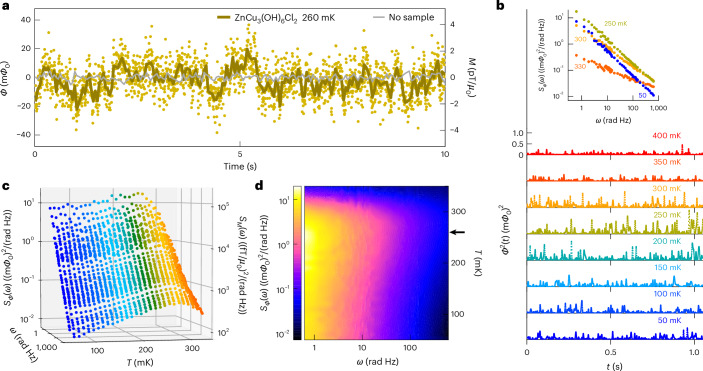
Spin noise spectroscopy of ZnCu_3_(OH)_6_Cl_2_. **a**, Typical time sequence of the measured magnetic flux Φ(t) generated by ZnCu_3_(OH)_6_Cl_2_ at 260 mK (light yellow dots), where frequency components out of bandwidth 0.3 rad Hz $\le \omega \le$ 600 rad Hz are filtered out. For visual clarity, the plotted data points are downsampled to 5 ms intervals. The boxcar average of the signal for every 50 ms is overlaid (dark yellow), which is highly distinct from the identically averaged signal of the empty coil (grey). **b**, Typical examples of the squared flux ${\varPhi }^{2}(t,T)$ at eight selected temperatures. Again, the signal bandwidth is 0.3 rad Hz $\le \omega \le$ 600 rad Hz and points are downsampled to 0.5 ms intervals. Inset: PSD ${S}_{\varPhi }(\omega ,T)$ at four selected temperatures after subtracting background contributions. The error bars are the standard error of segment averaging (see the ‘Measurements’ section). **c**, PSD ${S}_{\varPhi }(\omega ,T)$ of the measured ZnCu_3_(OH)_6_Cl_2_ witness spin flux noise, which spans a frequency range of at least 0.6 rad Hz $\le \omega \le$ 600 rad Hz. The spectra exhibit scale-invariant forms, ${\omega }^{-\alpha }$. The equivalent PSD ${S}_{M}(\omega ,T)$ of magnetization noise at the sample is presented in units of Tesla on the right axis. **d**, Contour plot of measured ${S}_{\varPhi }(\omega ,T)$ from **c**, revealing a clear transition in witness spin dynamics at ${T}^{* }\approx 260\,{\rm{mK}}$ (horizontal arrow). Source data

**Extended Data Fig. 2:**
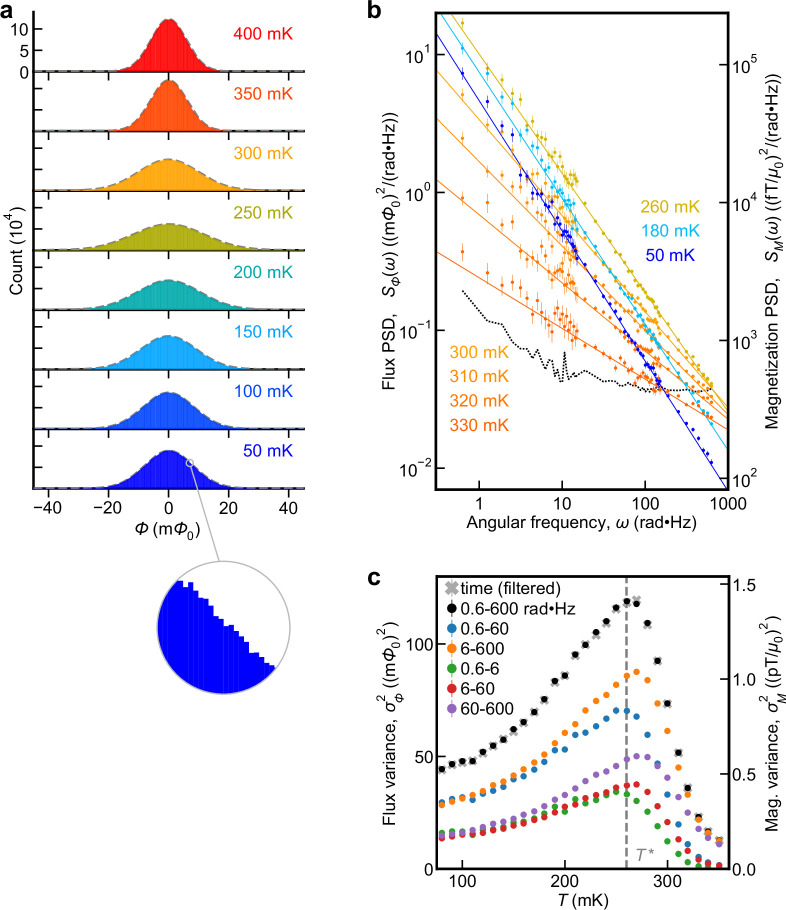
Analysis of ZnCu_3_(OH)_6_Cl_2_ spin noise spectroscopy. **a**. Typical distribution histograms of the measured ZnCu_3_(OH)_6_Cl_2_ witness spin flux noise $\varPhi \left(t,T\right)$ at eight selected temperatures (frequency components out of bandwidth 0.3 rad•Hz $\le \omega \le$ 600 rad•Hz are filtered out). Each histogram follows a Gaussian distribution (gray dashed line), with small statistical fluctuations (inset). **b**. Measured witness spin flux noise power spectral density ${S}_{\varPhi }\left(\omega ,T\right)$. Error bars are the standard error of segment averaging. The black dotted line indicates the measured background contribution that is subtracted from the signal. Lines represent $A\left(T\right){\omega }^{-\alpha \left(T\right)}$ fitting in the frequency range 0.6 rad•Hz $\le \omega \le$ 600 rad•Hz. **c**. Comparison of measured witness spin flux noise variance ${\sigma }_{\varPhi }^{2}$ obtained by direct calculation from $\varPhi \left(t,T\right)$ (after filtering) and by integrating ${S}_{\varPhi }\left(\omega ,T\right)$ in different frequency ranges. Error bars are propagated from power spectral density. Source data

Immediately on commencing our herbertsmithite experiments, we discovered that for $T < 400\,{\rm{mK}},$ all ZnCu_3_(OH)_6_Cl_2_ samples begin to spontaneously generate robust spin noise. As shown in Fig. 2a, the spin noise at $T=260\,{\rm{mK}}$ fluctuates with the maximum field amplitudes $B(t)\equiv {\mu }_{0}{M}(t)$ in the range of ${10}^{-12}\,{\rm{T}}$, orders of magnitude above the background. Figure 2b shows exemplary time sequences of the measured magnetic flux ${\varPhi }^{2}(t,T)$ for selected temperatures, demonstrating strong magnetization fluctuations that have Gaussian distributions (Extended Data Fig. 2a). From such data, we determine the power spectral densities (PSDs) of flux noise ${S}_{\varPhi }(\omega ,T)$ (Fig. 2b (inset) and Fig. 2c) for $0.6\,\mathrm{rad}\,\mathrm{Hz}\le \omega \le 600\,\mathrm{rad}\,\mathrm{Hz}$, with equivalent magnetic field noise on the right axis. Even at this elementary stage, herbertsmithite haecceity appears striking, because fluctuations in the spin-1/2 magnetization of a millimetre-scale sample are spontaneously generating magnetic fields near ${10}^{-12}$ T. Finally, Fig. 2d presents a colour-coded contour plot of measured ${S}_{\varPhi }(\omega ,T)$ revealing a clear transition in witness spin dynamics at ${T}^{\,* }\approx 260\,\mathrm{mK}$ (horizontal arrow). Since kagome spins have virtually no direct contribution to magnetic phenomena^12,13^ at subkelvin temperatures, and since the d.c. susceptibility χ(T) of our samples is quantitatively consistent with the magnetization expected when approximately 33% of Zn sites are occupied by Cu, the observed spin dynamics can only be explained by witness spin contributions.

**Fig. 3:**
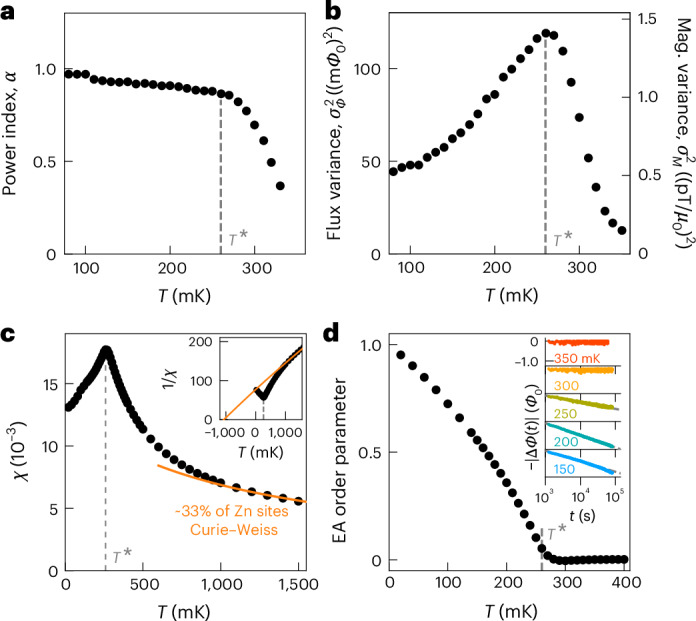
Spin noise spectroscopy analysis of witness spin dynamics in ZnCu_3_(OH)_6_Cl_2_. **a**, Measured witness spin flux noise power index α from ${S}_{\varPhi }(\omega ,T)\propto {\omega }^{-\alpha (T)}$ as a function of temperature (from Fig. 2c), obtained by fitting the PSD ${S}_{\varPhi }(\omega ,T)=A(T){\omega }^{-\alpha (T)}$ in the frequency range of 0.6 rad Hz $\le \omega \le$ 600 rad Hz. A clear transition to $\alpha \approx 1$ is detected at ${T}^{* }\approx 260\,{\rm{mK}}$ (dashed line). **b**, Measured witness spin flux noise variance ${\sigma }_{\varPhi }^{2}$ as a function of temperature (from Fig. 2c), obtained by integrating ${S}_{\varPhi }(\omega ,T)$ in the range of 0.6 rad Hz $\le \omega \le$ 600 rad Hz. Right axis: the equivalent magnetization noise variance ${\sigma }_{{M}}^{2}(T)$. These data indicate a transition in noise power at ${T}^{* }\approx 260\,{\rm{mK}}$ (dashed line). **c**, Measured witness spin susceptibility χ(T) (SI units; the inset shows 1/χ) in micro-Tesla magnetic fields revealing a Curie–Weiss-like χ(T) at higher temperatures (orange line), yielding an estimated witness spin density of 33% of Zn sites. However, this diverging \chi (T\right) is interrupted by a sharp transition to a rapidly diminishing χ(T) below ${T}^{* }\approx 260\,{\rm{mK}}$ (dashed line). **d**, Measured EA spin glass order parameter for witness spins from **c**, indicating a transition to a witness spin glass below ${T}^{* }\approx 260\,{\rm{mK}}$ (dashed line). Inset: time evolution of the average flux against a 2-μT applied field after the temperature is suddenly dropped from a thermalized condition at 400 mK. The sample flux Φ(t) shows a $-\mathrm{ln}(t)$ relaxation (dashed line) on periods of a day only below $T\approx 250\,{\rm{mK}}$, providing direct evidence of the appearance of a witness spin glass. The error bars are invisible as they are smaller than the symbol points in all panels. Source data

Fitting ${S}_{\varPhi }(\omega ,T)$, the spin noise spectra are found to be scale invariant. The measured witness spin noise power index α from fitting ${S}_{\varPhi }(\omega ,T)=A(T){\omega }^{-\alpha (T)}$ (Fig. 3a) undergoes a clear transition to $\alpha \approx 1$ at ${T}^{* }$ (vertical line). Simultaneously, the measured witness spin noise variance ${\sigma }_{\varPhi }^{2}(T)$, as determined by integrating ${S}_{\varPhi }(\omega ,T)$ in the range of 0.6 rad Hz $\le \omega \le$ 600 rad Hz (Fig. 3b) also plainly indicates a transition in the total noise power (vertical line). Thus, the herbertsmithite witness spin dynamics undergo a sharp transition at ${T}^{* }$.

**Extended Data Fig. 3:**
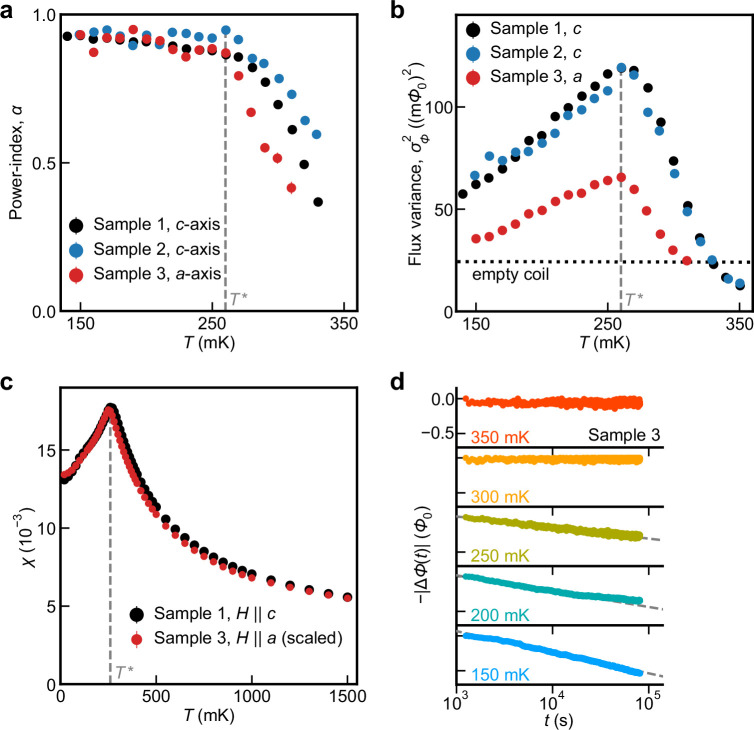
Comparison of spin noise and susceptibility measurements in multiple ZnCu_3_(OH)_6_Cl_2_ single crystals. **a**. Measured witness spin noise power-index α from ${S}_{\varPhi }\left(\omega ,T\right)\propto {\omega }^{-\alpha (T)}$ as a function of temperature. Error bars are the standard error from fitting. A clear transition to $\alpha \approx 1$ is detected at ${T}^{* }\approx 260\, {\rm{mK}}$ in all samples. **b**. Measured witness spin noise variance ${\sigma }_{\varPhi }^{2}$ as a function of temperature. A transition in noise power at ${T}^{* }\approx 260\, {\rm{mK}}$ (dashed line) is observed in all samples. The dotted line indicates the background noise variance. **c**. Measured witness spin susceptibility $\chi \left(T\right)$ (SI units) in micro-Tesla magnetic fields, showing a sharp transition to a rapidly diminishing $\chi \left(T\right)$ below ${T}^{* }\approx 260\, {\rm{mK}}$. The result of Sample 3 is scaled to facilitate a comparison. **d**. Time evolution of Sample 3 average flux $\varPhi \left(t\right)$ against a 2 μ${\rm{T}}$ applied field after a temperature quench from a thermalized condition at 400 mK, showing a $-\mathrm{ln}(t)$ relaxation (dashed line) on periods of a day only below ${T}^{* }\approx 260\, {\rm{mK}}$. The same behavior of Sample 1 is shown in the Fig. 3d inset. Source data

The witness spin magnetic susceptibility χ(T), measured in ultralow magnetic fields of $B\lesssim 4\,{\rm{\mu }}{\rm{T}},$ reveals the well-known^14,34^ Curie–Weiss behaviour of χ(T) at temperatures of *T* ≈ 1 K, quantitatively yielding that the witness spin density is $32.5 \% \pm 0.5 \%$ per plane of Zn^2+^ sites in our samples (see the ‘Herbertsmithite samples’ section). However, we find that this diverging χ(T) is first diverted from precise (${\chi }^{-1}\propto (T-{\theta }_{{\rm{CW}}})$) and then interrupted by a sharp transition to a rapidly diminishing χ(T) below ${T}^{* }$ (Fig. 3c). Although no such transition has been observed previously using higher magnitude fields^14^, we observe it in all samples (Extended Data Fig. 3). The combination of this cusp and noise transition suggests witness spin glass formation. Accordingly, we extract the Edwards–Anderson (EA) spin glass order parameter from these χ(T) data (see the ‘Measurements’ section). Although zero above ${T}^{* }$, it increases rapidly below (Fig. 3d). To confirm witness spin glass formation, we monitor the evolution of the flux Φ(t) in a 2-μ${\rm{T}}$ applied field under sudden thermal quenches from a thermalized condition at ${T}_{1}=400\,{\rm{mK}}$ to a lower temperature ${T}_{2}$ (Fig. 3d, inset). Here Φ(t) and, thus, the sample magnetization ${M}(t)$ enter a $-\mathrm{ln}(t)$ relaxation regime, requiring days to equilibrate, but this effect begins only when ${T}_{2} <$ ${T}^{* }.$ Together, these data provide direct confirmation of the appearance of a witness spin glass state at ${T}^{* }$ in ZnCu_3_(OH)_6_Cl_2_.

This begs the question of whether and how such witness spin interactions could be induced by a QSL. To address this, we consider a conventional Hamiltonian for mutual witness spin interactions as

1 $$H=\mathop{\sum }\limits_{ij}{J}_{{ij}}{{\boldsymbol{s}}}_{i}\cdot {{\boldsymbol{s}}}_{j},$$

where ${{\boldsymbol{s}}}_{i}$ is the spin-1/2 witness at lattice position *i*, the sum is over all pairs of such spins in the material and ${J}_{{ij}}$ represents the exchange energy scale between witness spins separated by ${r}_{{ij}}$. Here we consider a simple model of spinon-mediated interactions through a kagome Z_2_ QSL^35–39^, motivated in part by several earlier studies^40–42^. ${J}_{{ij}}$ may be estimated by modelling each witness spin as separately coupled to the three closest kagome spins in each of its two neighbouring planes (Fig. 1b, pyramidal links). In terms of the spin–spin susceptibility of the QSL linking the kagome spin sites *l* and *m* in the same plane, the witness spin interactions are

2 $${J}_{{ij}}=\frac{{\gamma }^{2}}{4}\mathop{\sum }\limits_{l\in {\therefore }_{i}}\mathop{\sum }\limits_{{m}\in {\therefore }_{j}}{\zeta }_{{lm}},$$

where γ is the strength of the witness spin to kagome coupling, ${\therefore }_{i}$ denotes the three kagome sites nearest to witness spin *i* (Fig. 1b, pyramidal links), and ${\zeta }_{{lm}}$ is the static spin susceptibility between kagome sites *l* and *m* mediated by the QSL. To estimate ${\zeta }_{{lm}}$, we evaluate the spinon band structure using a Schwinger fermion mean field decoupling of the kagome Heisenberg antiferromagnet^36^. Although not as precise as density matrix renormalization group or exact diagonalization, the model allows us to reach large system sizes necessary for long-range interactions. For our QSL Hamiltonian ${H}_{{\rm{QSL}}}$, the parameters are the nearest-neighbour spinon hopping rate ${t}_{1}/\hslash$, the second-neighbour rate ${t}_{2}/\hslash$, the gap parameter, and two Lagrange multipliers which enforce the physical Hilbert space constraint of half-filling. We determine all parameters self-consistently for the kagome plane of herbertsmithite, yielding a gap of $2\Delta \approx 0.44{t}_{1}\approx 30\,{\rm{K}}$ consistent with previous theoretical studies^35,37,39^ (see the ‘Spinon-mediated interactions via Z_2_ QSL’ section) and the experimental kagome-lattice susceptibility ${\chi }_{{\rm{K}}}(T)$ collapsing for $T\lesssim 30\,{\rm{K}}$ (ref. ^16^). Linear response theory for the gapped parabolic spinon band structure of QSL yields an intra-kagome spin susceptibility of

3 $${\zeta }_{{lm}}=-\frac{2}{\pi }{\int }_{\!\!-\infty }^{{E}_{{\rm{F}}}}{\mathfrak{I}}[{G}_{{lm}}(E){G}_{{ml}}(E)]{\rm{d}}E,$$

where

4 $${G}_{lm}(E)=\langle l|\left(E+i\eta -{H}_{\rm{QSL}}\right)^{-1}|m\rangle$$

is the real-space spinon Green’s function connecting sites *l* and *m* at energy *E*, whereas *η* is an infinitesimal positive regularization. We evaluate ${\zeta }_{{lm}}$ using exact diagonalization on clusters of up to 20 × 40 kagome unit cells. We find good agreement with approximate analytic results obtained for the similar case of electron-mediated interactions in gapped graphene^41,42^ (where the parabolic electron bands are due to spin–orbit interactions) in the long-distance limit:

5 $${J}_{{ij}}\left(r\right)\propto \frac{\exp \left(-\frac{r}{{r}_{0}}\right)}{{r}^{\frac{3}{2}}},$$

where *r* is the separation of witness spins *i* and *j*, and for the Z_2_ QSL we would expect

6 $$\,{r}_{0}=\sqrt{2}d\left(\frac{{t}_{1}}{2\Delta }\right),$$

where *d* is the nearest-neighbour kagome Cu spacing. Crucially, when spinon mediated, we find that all witness spin interactions are purely AF. This is consistent with the AF nearest-neighbour interaction associated with the spin structure factor from inelastic neutron scattering studies^17^, simultaneously generating further neighbour interactions that frustrate the witness spin interactions. Overall, this reveals how the gapped spinon spectrum of a Z_2_ QSL in herbertsmithite could robustly mediate interactions between quantum witness spins.

One free parameter *γ* controls the intensity of spinon mediation of witness spin dynamics. We constrained γ by the accepted Curie–Weiss temperature ${\theta }_{{\rm{CW}}}=-1.1\,{\rm{K}}$ (see the ‘Spinon-mediated interactions via Z_2_ QSL’ section), yielding $\left|\gamma \right|=60\,{\rm{K}}\approx {J}_{{\rm{K}}}/3$. We note that both ferromagnetic and AF coupling $\gamma =\pm 60\,{\rm{K}}$ are consistent with our model described by ${\left|\gamma \right|}^{2}$. Further, the order of magnitude of $\left|\gamma \right|$ seems plausible because the witness spins and the kagome spins are both Cu^2+^ s=1/2 ions with similar Cu–Cu separations (although their bond angles with the oxygen atoms do differ, and hence, |γ| could have been much smaller)^43^. However, this brings into sharp focus a key mystery in herbertsmithite studies: virtually, all low-temperature spin dynamical phenomena^14,15,17,25–27,34^ occur approximately two orders of magnitude lower in temperature than ${J}_{{\rm{K}}}$. Most obviously, why is ${\theta }_{{\rm{CW}}}$ so strongly suppressed below the natural Cu^2+^ s=1/2 interaction scale ${J}_{{\rm{K}}}$? Spinon mediation provides a simple and quantitative explanation: nearest-neighbour witness spin interactions communicate via spinons propagating through the QSL at the third order. The estimated intra-kagome spin susceptibility in the dimensionless form, ${\zeta }_{{lm}}^{{\,\prime} }\approx {t}_{1}{\zeta }_{{lm}}$, then reveals from equation (2) that the spinon-mediated witness spin interaction energy scale is

7 $$\frac{{\gamma }^{2}}{4}\frac{{\zeta }_{{lm}}^{{\,\prime} }}{{t}_{1}}\approx \frac{{\left(60\,{\rm{K}}\right)}^{2}}{4}\frac{0.1}{76\,{\rm{K}}}\,\approx 1.2\,{\rm{K}}$$

because for the herbertsmithite Z_2_ QSL theory, equation (3) yields ${\zeta }_{{lm}}^{{\,\prime} }\approx 0.1$ for the nearest neighbours and ${t}_{1}=0.4{J}_{{\rm{K}}}\approx 76\,{\rm{K}}$ (ref. ^28^). This agrees strikingly with the experimental value |${\theta }_{{\rm{CW}}}|\approx 1.1\,{\rm{K}}$. Thus, the energy scale of a wide variety of T→0 spin dynamical characteristics^14,15,17,25–27,34^ of herbertsmithite emerges naturally and quantitatively from the spinon mediation of witness spin dynamics.

**Fig. 4:**
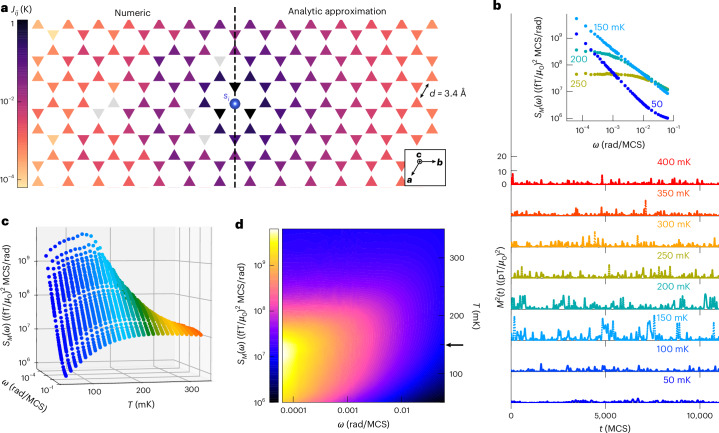
Simulated spinon-mediated witness spin noise dynamics in ZnCu_3_(OH)_6_Cl_2_. **a**, Spinon-mediated interaction energy ${J}_{{ij}}(r)$ between witness spins. Left: numerically calculated interactions due to a single Cu^2+^ witness spin at the origin (equation (2)). The grey colour indicates ${J}_{{ij}}(r) < 0$. Right: analytic long-distance approximation (equation (5)). **b**, Typical example of the simulated magnetization dynamics ${{M}}(t)$ of witness spins under extensive spinon-mediated exchange interactions, plotted as ${{{M}}}^{2}(t,T)$, at eight selected temperatures. Frequency components out of bandwidth $3\times {10}^{-5}$ rad/MCS $\le \omega \le$ $6\times {10}^{-2}$ rad/MCS are filtered out, and points are downsampled to 5 MCS intervals. Inset: PSD ${S}_{{{M}}}(\omega ,T)$ at four selected temperatures. The error bars are the standard error of segment averaging (see the ‘Witness spin Monte Carlo simulations’ section). **c**, Predicted PSD ${S}_{{{M}}}(\omega ,T)$ of witness spin magnetization noise as a function of frequency and temperature due to spinon-mediated interactions. **d**, Contour plot of predicted ${S}_{{{M}}}(\omega ,T)$ from **c** revealing a clear transition in dynamics at ${T}^{* }\approx 150\,{\rm{mK}}$ (horizontal arrow). Source data

**Fig. 5:**
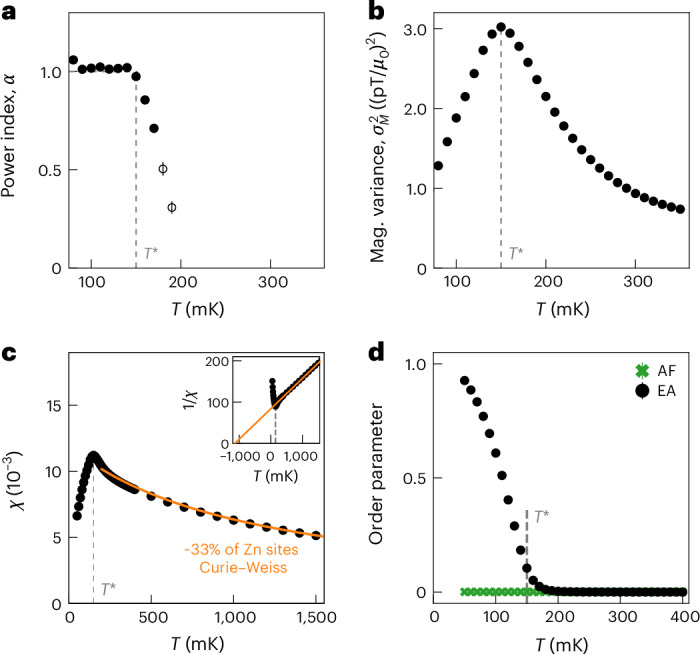
Spectroscopy analysis of simulated spinon-mediated witness spin dynamics in ZnCu_3_(OH)_6_Cl_2_. **a**, Predicted witness spin magnetization noise power index α for ${S}_{{{M}}}(\omega ,T)\propto {\omega }^{-\alpha (T)}$ as a function of temperature (from Fig. 4c), revealing a transition to $\alpha \approx 1$ at ${T}^{* }$ (dashed line). The open circles are used for temperatures above ${T}^{* }$ where power-law fitting is challenging. The error bars are the standard error from fitting. **b**, Predicted witness spin magnetization noise variance ${\sigma }_{{{M}}}^{2}$ as a function of temperature (from Fig. 4c), indicating a transition in noise power at ${T}^{* }$ (dashed line). **c**, Predicted witness spin-only susceptibility χ(T) (SI units; the inset shows 1/χ) due to spinon-mediated interactions, revealing a sharp transition at ${T}^{* }$ (dashed line) from a Curie–Weiss behaviour determined at higher temperatures (orange line). **d**, Predicted AF order parameter (green crosses) and EA spin glass order parameter (black dots) from witness spin simulations, indicating that ${T}^{* }$ (dashed line) is the transition to a spinon-mediated witness spin glass. The error bars are invisible as they are smaller than the symbol points in **b**–**d**. Source data

**Extended Data Fig. 4:**
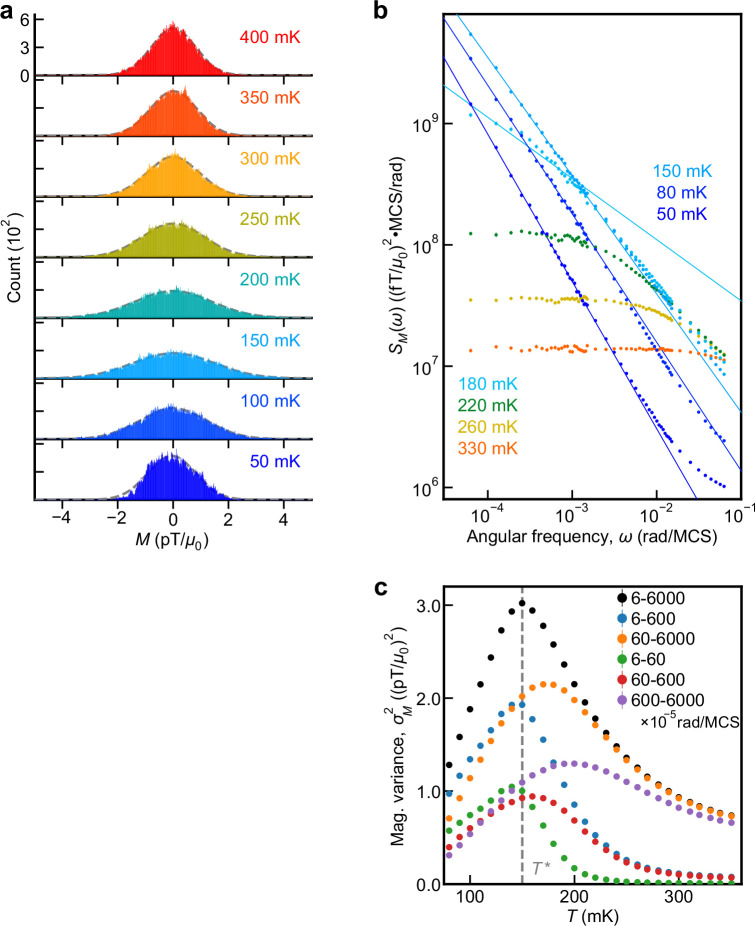
Analysis of simulated spinon-mediated witness spin noise spectroscopy. **a**. Typical distribution histograms of the simulated witness spin magnetization noise ${{M}}\left(t,T\right)$ at eight selected temperatures (frequency components out of bandwidth $3\times {10}^{-5}$ rad/MCS $\le \omega \le$ $6\times {10}^{-2}$ rad/MCS are filtered out). Each histogram follows a Gaussian distribution (gray dashed line) with small statistical fluctuations. **b**. Predicted witness spin magnetization noise power spectral density ${S}_{{{M}}}\left(\omega ,T\right)$. Error bars are the standard error of Monte-Carlo-run averaging. Lines represent $A\left(T\right){\omega }^{-\alpha \left(T\right)}$ fitting in the frequency range $6\times {10}^{-5}$ rad/MCS $\le \omega \le$ $1\times {10}^{-3}$ rad/MCS. **c**. Comparison of simulated witness spin magnetization noise variance ${\sigma }_{{{M}}}^{2}$ obtained by integrating ${S}_{{{M}}}\left(\omega ,T\right)$ in different frequency ranges. Source data

**Extended Data Fig. 5:**
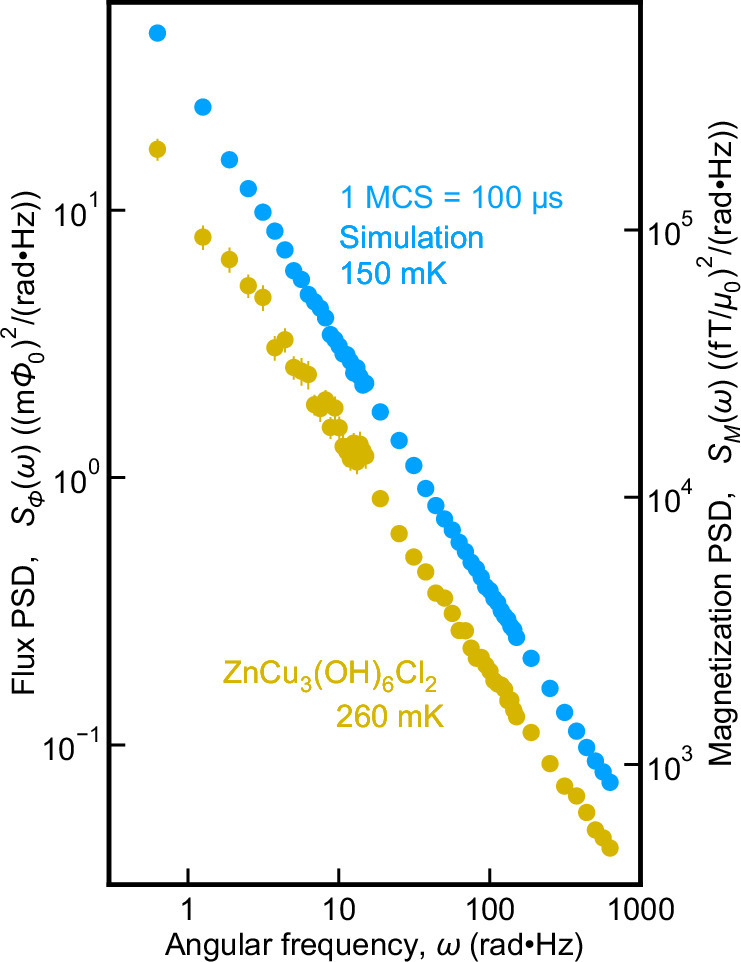
Witness spin noise power spectral density when 1 Monte Carlo step = 100 μs. Simulated witness spin magnetization noise power spectral density ${S}_{{{M}}}\left(\omega \right)$ at ${T}^{* }$ (blue), when 1 Monte Carlo step is set to 100 μ${\rm{s}}$. It falls into the frequency window of measured ZnCu_3_(OH)_6_Cl_2_ witness spin noise at ${T}^{* }$ (yellow): 0.6 rad•Hz $\le {\omega} \le$ 600 rad•Hz. Error bars are the standard error of segment averaging. Source data

To understand the global consequences requires the large-scale simulations of spinon-mediated witness spin dynamics. Accordingly, we simplified equation (1) by approximating the quantum spins with classical Ising variables, enabling us to conduct Monte Carlo (Metropolis–Hastings) simulations. The validity of this approximation for use in herbertsmithite witness spin dynamics simulations is discussed in detail in the ‘Witness spin Monte Carlo simulations’ section. Each witness spin interacts with all others that share a kagome plane. We use our numerically calculated ${J}_{{ij}}(r)$, equation (2), with 45 × 45 × 4 possible witness spin sites (randomly occupied to ~33% per Zn^2+^ plane) and periodic boundary conditions (see the ‘Witness spin Monte Carlo simulations’ section). We present the interaction energy ${J}_{{ij}}(r)$ between a single witness spin at the centre and one at any other witness spin site, along with the analytic approximation (Fig. 4a). An important consequence stemming from ${J}_{{ij}}(r)$ is that although the known nearest-neighbour AF witness spin interactions would lead to AF order (above the percolation threshold), the extensive spinon-mediated interactions frustrate this ordering. This generates a plethora of physical effects. Most elementary is the time sequence of predicted magnetization fluctuations ${{M}}(t,T)\propto {\sum }_{i}{s}_{i}(t,T)$, where *i* represents all witness spin sites in the simulated crystal. The fluctuations in witness spin ${{M}}(t,T)$ are predicted to intensify and slow as falling ${k}_{{\rm{B}}}T$ approaches ${J}_{{ij}}(r=d){s}_{i}{s}_{j}$ (Fig. 4b). The fluctuations have Gaussian distributions (Extended Data Fig. 4a). The predicted PSD of this witness spin magnetization noise ${S}_{{{M}}}(\omega ,T)$ (measured in units of radians/MCS where MCS is the Monte Carlo time step) is shown in Fig. 4b (inset) and Fig. 4c, whereas Fig. 4d shows the contour plot of ${S}_{{{M}}}(\omega ,T)$ predictions revealing a transition in witness spin dynamics at ${T}^{* }\approx 150\,{\rm{mK}}$ (horizontal arrow). Further, the witness spin noise power index α(T) derived from fitting ${S}_{{{M}}}(\omega ,T)\propto {\omega }^{-\alpha (T)}$ to the data in Fig. 4c,d transitions to a constant $\alpha \approx 1$ at ${T}^{* }$ (Fig. 5a) (see the ‘Witness spin Monte Carlo simulations’ section). The witness spin noise variance ${\sigma }_{{{M}}}^{2}(T)$ from Fig. 4c,d exhibits a transition in noise power at ${T}^{* }$ below which it collapses (Fig. 5b). Moreover, although the d.c. magnetic susceptibility of the witness spins approximately follows a Curie–Weiss trajectory at higher temperatures near $T\approx 1\,{\rm{K}}$ (Fig. 5c), once the spinon-mediated interactions become predominant, the Z_2_ QSL model predicts a sharp cusp in χ(T) at a transition temperature near $150\,{\rm{mK}}$ (Fig. 5c). Finally, analysis using all the predicted witness spin configurations ${s}_{i}$ in terms of either an AF or EA spin glass order parameter (Fig. 5d) reveals the finite EA (and zero AF) order parameter, indicating witness spin freezing. By juxtaposing the diverse predictions from equations (1)–(4) for spinon-mediated witness spin dynamics and the ground state (Fig. 5), with the discovered witness spin dynamical phenomenology of herbertsmithite (Fig. 3), their conspicuous correspondence evidently validates the spinon-mediated witness spin dynamics concept (see also Extended Data Fig. 5).

**Extended Data Fig. 6:**
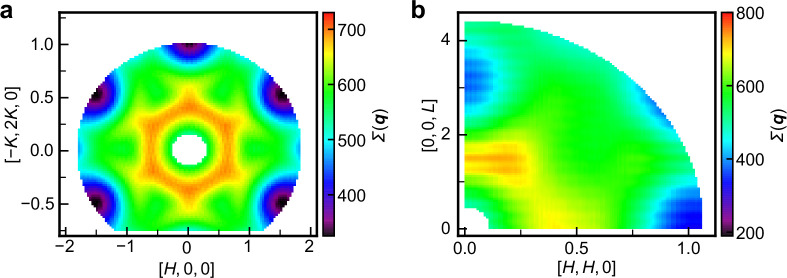
Simulated witness spin structure factor. The witness spin structure factor ${\Sigma} \left({\boldsymbol{q}}\right)$ that is calculated from a spin-configuration snapshot in the Monte Carlo simulation at 2 K for (*HK*0) space (**a**) and (*HHL*) space (**b**). Source data

This revelation resolves open mysteries of the T→0 phenomenology of herbertsmithite. Although Cu^2+^/Zn^2+^ substitution has long been adduced as the trigger for such effects^6–8,11–20,22–27,34,44–53^, no specific microscopic theory has previously been put forward. Indeed, the opposite approach has been more typical, with much effort expended in minimizing their influence on results. Here we explore the obverse perspective: the identification of a specific microscopic mechanism and its quantitative theory for witness spin interactions via the QSL. Then, we find the susceptibility ${\chi }^{-1}(T)\propto (T-{\theta }_{{\rm{CW}}})$ with ${\theta }_{{\rm{CW}}}\approx -1.1\,{\rm{K}}$ is highly consistent with the spinon-mediated ${J}_{{ij}}(r)$ of the witness spin interactions (Fig. 5c). Similarly, we confirm (see the ‘Neutron scattering structure factor’ section and Extended Data Fig. 6) that the low-energy neutron scattering structure factor ${\Sigma} ({\boldsymbol{q}},\omega < 1\,\mathrm{meV})$ of herbertsmithite^17,26^ is not inconsistent with witness spin dynamics controlled by the spinon-mediated ${J}_{{ij}}(r)$ between pairs of nearest-neighbour witness spins (Fig. 4a). With respect to ^17^O nuclear magnetic resonance relaxation rates, $1/{T}_{1}(T)$, their sharp diminution below $T\approx 250\pm 50\,{\rm{mK}}$ (ref. ^47^) is consistent with the suppression of witness spin fluctuations below ${T}^{* }$, as predicted within our spinon mediation theory (Fig. 5b).

Our empirical knowledge of herbertsmithite witness spin interactions with its QSL has thus been augmented and clarified by the introduction of spin noise spectroscopy^29–33^ to QSL studies. This reveals the existence (Fig. 2a,b), slowing and intensification of scale-invariant witness spin noise with PSD $S(\omega ,T)\propto {\omega }^{-\alpha \left(T\right)}$ (Fig. 2b,c,d). These fluctuations evolve to reach a constant $\alpha \approx 1$ at ${T}^{* }\approx 260\,{\rm{mK}}$ (Fig. 3a), at which point the spin noise power ${\sigma }_{{{M}}}^{2}(T)$ begins to diminish steeply (Fig. 3b). Moreover, at this ${T}^{* }$, the susceptibility χ(T) (measured at previously unexplored micro-Tesla fields) experiences a sharp transition into a witness spin glass phase (Fig. 3c), exhibiting an EA spin glass order parameter and ultraslow relaxation (Fig. 3d). All these phenomena are consistent within a model having a $2\Delta \approx 0.44{t}_{1}\approx 30\,{\rm{K}}$ gapped spinon spectrum of a Z_2_ QSL in herbertsmithite (see the ‘Spinon-mediated interactions via Z_2_ QSL’ section), thereby explaining the T→0 phenomenology if the coupling constant between Cu^2+^ witness spins and kagome spins is $\left|\gamma \right|\approx {J}_{{\rm{K}}}/3$. The form and range of the witness spin interaction function ${J}_{{ij}}(r)$ (Fig. 4a) and, more fundamentally, the quantitative form and structure of the spin–spin susceptibility within the Z_2_ QSL, are then determined theoretically.

**Extended Data Fig. 7:**
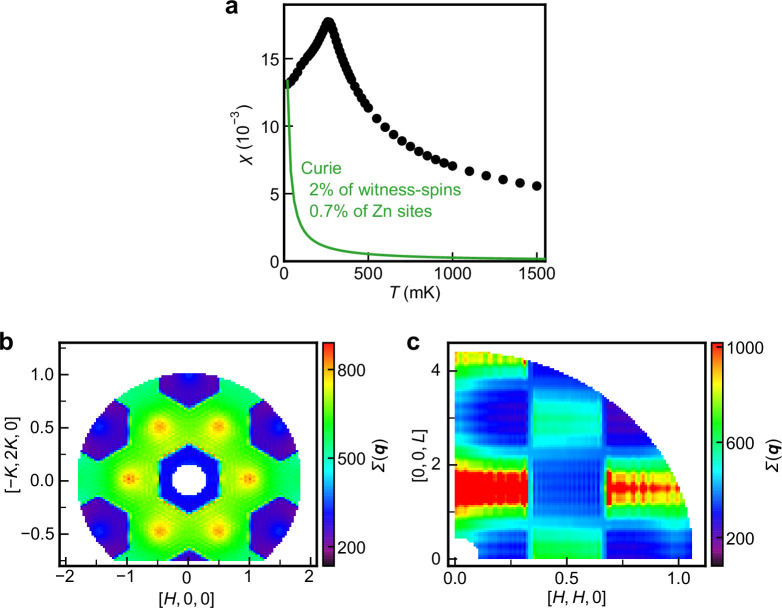
Observations constraining alternative theoretical models. **a**. The estimated isolated-spin magnetic susceptibility ${\chi} (T)$ of 2% of witness spins (0.7% of Zn sites) overlayed on the measured witness spin susceptibility of Fig. 3c (black dots). **b,c**. The witness spin structure factor ${\Sigma} \left({\boldsymbol{q}}\right)$ assuming spin-wave mediated interactions that decay as $1/r{\sim} 1/{r}^{2}$. It is calculated from a snapshot of witness spin configuration in the Monte Carlo simulation at 2 K for (*HK*0) space (**b**) and (*HHL*) space (**c**). The quantitative detail is dependent on the in-plane distance cutoff due to the slow decay of the interaction, and here a 45x45 cutoff is used. Source data

**Extended Data Fig. 8:**
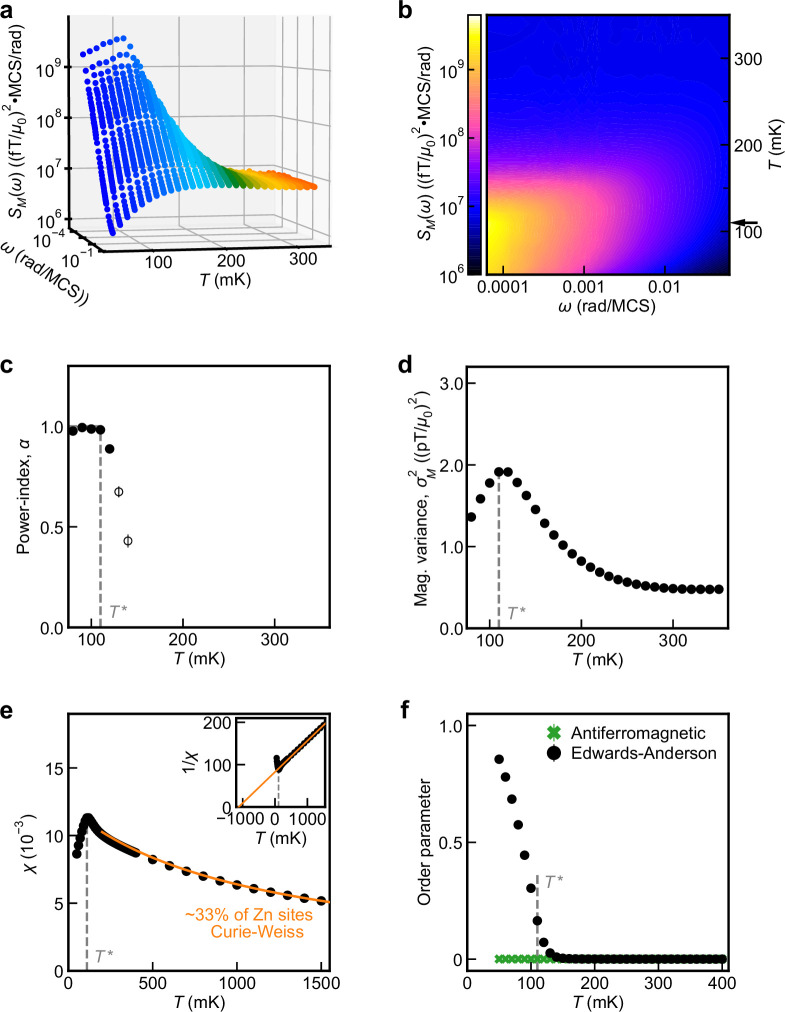
U(1) quantum spin liquid spinon-mediated witness spin dynamics in ZnCu_3_(OH)_6_Cl_2_. **a**. Predicted power spectral density of witness spin magnetization noise ${S}_{{{M}}}\left(\omega ,T\right)$ as a function of frequency and temperature due to spinon-mediated interactions. **b**. Contour plot of ${S}_{{{M}}}\left(\omega ,T\right)$ from **a** revealing a clear transition in dynamics at ${T}^{* }\approx 110\,{\rm{mK}}$ (horizontal arrow). **c**. Predicted witness spin magnetization noise power-index α for ${S}_{{{M}}}\left(\omega ,T\right)\propto {\omega }^{-\alpha (T)}$ as a function of temperature from **a**, revealing a transition to $\alpha \approx 1$ at ${T}^{* }$ (dashed line). The open circles are used for temperatures above ${T}^{* }$ where a power-law fitting is challenging. Error bars are the standard error from fitting. **d**. Predicted witness spin magnetization noise variance ${\sigma }_{{{M}}}^{2}$ as a function of temperature from **a**, indicating a transition in noise power at ${T}^{* }$ (dashed line). **e**. Predicted witness-spin-only susceptibility $\chi \left(T\right)$ (SI units, inset shows 1/χ) due to spinon-mediated interactions, revealing a sharp transition at ${T}^{* }$ (dashed line) from a Curie-Weiss behaviour determined at higher temperatures (orange line). **f**. Predicted antiferromagnetic order parameter (green crosses) and Edwards-Anderson spin-glass order parameter (black dots) from witness spin simulations, indicating that ${T}^{* }$ (dashed line) is a spin-freezing transition. Source data

We have considered a range of alternative models, including random spin singlets, Zn substitution into the kagome planes, witness spin dipole–dipole interactions, spin-wave-mediated witness spin interactions, ferromagnetic clusters and local magnetic exchange mechanisms, and all of them can be ruled out based on the experimentally determined phenomenology as T→0, as comprehensively explained in the ‘Considering alternatives to spinon-mediated witness spin interactions’ section and Extended Data Fig. 7. Only spinon mediation shows consistency with the full range of experimental data (Fig. 3), and that of a Z_2_ QSL (Fig. 5) gave a somewhat better quantitative match than a U(1) QSL (Extended Data Fig. 8). However, it remains challenging to give a definitive discrimination between Z_2_ and U(1) spinon mediation of witness spin interactions. A comprehensive fully quantum mechanical theory for the influences of witness spins on the kagome plane and vice versa may be required. This could greatly improve the precision of the theoretical model, for example, the spin noise spectral shape above ${T}^{* }$ and the detailed spin dynamics as T→0 (see the ‘Witness spin Monte Carlo simulations’ and ‘Neutron scattering structure factor’ sections). Such a study might also quantify the extent to which witness spins could affect the quantum state in the kagome plane, further enhancing the efficacy of the witness spin approach to probing QSLs.

Notwithstanding these theoretical challenges, further exceptional opportunities for QSL studies also emerge. First, Cu^2+^/Zn^2+^ substitutional atoms no longer obscure the QSL physics of herbertsmithite, but, instead, they would potentially allow the direct quantum detection and interrogation of the spinon spectrum (Figs. 3 and 5). Second, the herbertsmithite witness spin glass state as T→0 appears to be a unique state of quantum matter, predicated on long-range quantum entanglement through a QSL. Third, witness spins may now be used as a ‘quantum portal’ through which to access, manipulate and transit the QSL. Finally, these witness spin noise techniques and interpretations are eminently generalizable to wide-ranging QSL research in other target materials (for example, Zn barlowite), as well as to similarly elusive states such as random-bond quantum dimer systems.

## Methods

### Herbertsmithite samples

ZnCu_3_(OH)_6_Cl_2_ single crystals were synthesized as described in ref. ^54^, using a recrystallization method. Powders of ZnCl_2_, CuO and H_2_O were mixed in a quartz tube with a ratio of 2.015 g:0.235 g:4.5 ml. The tube was sealed under vacuum and laid horizontally in a three-zone gradient furnace, with the temperature of hot and cold ends set at 180 °C and 160 °C, respectively. Millimetre-scale single crystals were obtained after 3 months. Extended Data Fig. 1a shows the photographs of the three ZnCu_3_(OH)_6_Cl_2_ single crystals studied. The lattice structure was confirmed by X-ray Laue diffraction, as exemplified by the clear Bragg peaks of Sample 1′ (Extended Data Fig. 1b). Measurements were performed along the *c* axis for Samples 1 and 2 and the *a* axis for Sample 3.

The stoichiometry of Zn:Cu ratio is found to be 0.97:3.03 for the samples reported here, by using inductively coupled plasma mass spectrometry. The refinement of a single-crystal X-ray diffraction measurement indicates that 32.5% of the Zn^2+^ sites and 10.8% of the Cu^2+^ sites are inter-substituted^24^. Extended Data Fig. 1c shows the d.c. susceptibility (Sample 1′) in SI units measured by a SQUID magnetic property measurement system (Quantum Design) and its Curie–Weiss fitting by $\chi =\,{\chi }_{0}+\frac{{C}_{{\rm{Curie}}}}{T-{\theta }_{{\rm{CW}}}}$. Fitting in a temperature range of 150 K $\le T\le$ 320 K yields ${\chi }_{0}=-5\times {10}^{-6}$, ${\theta }_{{\rm{CW}}}=-280$ K and ${C}_{{\rm{Curie}}}=0.165$ K. Fitting in a temperature range of 2 K $\le T\le$ 6 K yields ${\chi }_{0}=(4.3\pm 0.2)\times {10}^{-4}$, ${\theta }_{{\rm{CW}}}=-1.07\pm 0.03\;{\rm{K}}$  and ${C}_{{\rm{Curie}}}=0.0134\pm 0.0002\;{\rm{K}}$  corresponding independently to *S* = 1/2 at 32.5% ± 0.5% of the Zn sites. The Curie–Weiss fitting of the low-temperature d.c. susceptibility is stable as long as the fitting range is within 2 K $\le T\le 10$ K, although it becomes sharply fitting range dependent below 1 K at which the susceptibility starts diverting from the Curie–Weiss behaviour (Fig. 3c)^14^. These sample characterization results are comparable with past stoichiometry studies^11,19,44^, neutron diffraction studies^11,19,55^ and d.c. susceptibility studies^14,34,56^.

As the Cu occupation probability of witness spin sites in our single crystals, we take 33%. This value is based on the coincidence of estimates from the single-crystal X-ray diffraction measurement^24^ and d.c. susceptibility Curie–Weiss fitting (Extended Data Fig. 1c), both of which are performed on our single crystal (Sample 1′). We note that the precise nature of site disorder in herbertsmithite has not yet been fully determined^11,22,23,57^, with reported Cu substitution percentage at Zn sites ranging from 12% (ref. ^17^) to 36% (ref. ^55^), and of the Zn substitution percentage at Cu ranging from 0% (refs. ^11,57^) to 10% (refs. ^22,55^).

### Measurements

Magnetic flux Φ(t) noise measurements were performed with a 19-turn single superconducting pickup coil connected to a d.c. SQUID SQ1200 (Star Cryoelectronics), which is designed to maximize the noise measurement sensitivity. Susceptibility measurements were performed with a 10-turn-each in-series counter-wound superconducting pickup coil connected to a SQUID SP550 (Quantum Design) (Fig. 1c), using a solenoid whose magnetic field was calibrated by using an indium cylinder in a superconducting state ($\chi =\,-1$). In both setups, three 0.2-mm-diameter silver wires were directly attached to the sample for thermalization, and measurements were taken at least 20 min after the target temperature was reached. Both setups were shielded by multiple nested niobium and mu-metal cylinders.

Flux noise data at each temperature was recorded for 1,000 s at 20 kSa s^−1^. For susceptibility measurements at each temperature, the sample magnetic response was recorded as the magnetic field ${\mu }_{0}H$ was swept over $0\,{\rm{\mu }}{\rm{T}}\to \,-4\,{\rm{\mu }}{\rm{T}}\to 4\,{\rm{\mu }}{\rm{T}}\to -4\,{\rm{\mu }}{\rm{T}}\to 0\,{\rm{\mu }}{\rm{T}}$ in steps of $\sim 0.05\,{\rm{\mu }}{\rm{T}}$ (zero-field cooling). The slope of these $({\mu }_{0}{{M}})/({\mu }_{0}H)$ data, representing the magnetic susceptibility, is extracted by a linear fitting with standard error bars from the linear fit. The micro-Tesla d.c. susceptibility of Sample 1 (Fig. 3c) is obtained by subtracting an offset constant. This offset value is determined so that the measured micro-Tesla susceptibility smoothly connects to the measurement result in the magnetic property measurement system in the overlapping temperature range of 2 K ≤ *T* ≤ 3 K (Extended Data Fig. 1d). For the long-term spin evolution under a 2-μT field, a sample was thermalized at ${T}_{1}=400\,{\rm{mK}}$ for 1 h and the temperature was rapidly dropped to a lower temperature ${T}_{2}$ in less than 5 min. After 20 min of thermalization at ${T}_{2}$ (that is, starting from t= 1,200 s), the spin evolution was recorded for 80,000 s (~1 day) at 1 kSa s^−1^. In Fig. 3d (inset), the signal is averaged for every 100 s.

Flux noise data are processed in a similar method as that in ref. ^32^. The distribution of Φ(t) is Gaussian with the expected statistical fluctuations (Extended Data Fig. 2a). The PSD with frequency resolution $\Delta \omega =\left(2{\rm{\pi }}\,{\rm{rad}}\right)\times \left(0.1\,{\rm{Hz}}\right)=0.6$ rad Hz is first calculated from 100 split segments^32^, with its error bars determined by the standard error of segment averaging. The empty-coil measurement result is subtracted as a background contribution. This PSD is plotted in Fig. 2c,d and Extended Data Fig. 2b. To increase the signal-to-noise ratio, the PSD is averaged over a 10$\Delta \omega$ or $100\Delta \omega$ window at high frequencies. The power-law index shown in Fig. 3a is obtained by fitting the $\Delta \omega =0.6$ rad Hz PSD with ${S}_{\varPhi }(\omega ,T)\propto {\omega }^{-\alpha (T)}$ in the range of 0.6 rad Hz $\le \omega \le$ 600 rad Hz (Extended Data Fig. 2b). The error bars are the standard error from fitting. The variance ${\sigma }_{\varPhi }^{2}$ shown in Fig. 3b is calculated by integrating the $\Delta \omega =0.6$ rad Hz PSD in the range of 0.6 rad Hz $\le \omega \le$ 600 rad Hz (error bars are propagated from the PSD). This is demonstrably equivalent to the variance directly calculated from the time sequence of Φ(t) (appropriately filtered to the corresponding frequency range), and the variance peak temperature remains close to ${T}^{* }$ for all different frequency-integration ranges (Extended Data Fig. 2c).

The EA spin glass order parameter ${q}_{{\rm{EA}}}$ shown in Fig. 3d is extracted from the micro-Tesla d.c. susceptibility by solving the formula^58,59^

8 $${\chi} (T)=\frac{C\left(1-{q}_{{\rm{EA}}}(T)\right)}{T-\theta \left(1-{q}_{{\rm{EA}}}(T)\right)}.$$

The constants C and θ are obtained by fitting in the temperature range above ${T}^{* }$ for which ${q}_{{\rm{EA}}}(T)$ vanishes: 270 mK $\le T\le$ 500 mK. The error bars of ${q}_{{\rm{EA}}}(T)$ propagate from χ(T) and the standard error of the fitted parameters C and θ.

The spin noise data shown in Figs. 2 and 3 were measured in Sample 1. The equivalent measurements were performed for Sample 2 and Sample 3. As shown in Extended Data Fig. 3, the transition at $T^{*} \approx 260\,\mathrm{mK}$ in the witness spin noise power index α from the PSD ${S}_{\varPhi }\left(\omega ,T\right)\propto {\omega }^{-\alpha (T)}$, the witness spin noise variance ${\sigma }_{\varPhi }^{2}$ and the micro-Tesla susceptibility χ; and the $-\mathrm{ln}(t)$ relaxation of the sample flux $\varPhi \left(t\right)$ below ${T}^{* }$ are reproduced in multiple samples. The Sample 3 response was smaller compared with the other two samples because the small crystal size made it difficult to fill the full length of the pickup coil. Accordingly, the PSD fitting range is limited to 0.6 rad Hz $\le \omega \le$ 60 rad Hz and the susceptibility measurement result in Extended Data Fig. 3c is scaled for comparison with Sample 1.

The witness spin dynamics and the associated transition that we observe in all herbertsmithite samples had not been previously measured in either a.c. or d.c. susceptibility studies^14,44^. One possible reason could be a difference in measurement conditions. Pioneering d.c. susceptibility measurements^14^ were performed at magnetic fields near B= 0.05 T. Although that field is small compared with the energy scale of the observed transition temperature ${T}^{* }\approx 260\,\mathrm{mK}$, it is empirically known that fairly a small field can considerably suppress a sharp peak signature of spin glass transition, making it difficult to detect. For example, a d.c. field of B= 0.04 T is capable of suppressing the sharp peak signature of a 21.5-K spin glass transition in Fe_0.5_Mn_0.5_TiO_3_ (ref. ^60^), 0.05 T for a 17-K transition in CdCr_1.7_In_0.3_S_4_ (ref. ^61^), 0.06 T for a 15.5-K transition in Gd_0.37_Al_0.63_ (ref. ^62^) and 0.04 T for a 0.2-K transition in Gd_3_Ga_5_O_12_ (ref. ^63^). Another possible reason is the difference between powder samples and single crystals. The herbertsmithite susceptibility studies^14,44^ were performed on powder samples before the establishment of single-crystal growth^54^. Differences between single crystal and such powder samples might possibly be caused by different chemical compositions, enhanced surface effects and randomized direction of applied field, which may have prevented the observation of a spin glass transition therein. All of the d.c. susceptibility measurements reported in the present work were carried out at $B\le 5\,{\rm{\mu }}{\rm{T}}$ on single crystals, and all of them yield a sharp transition at a virtually identical ${T}^{* }\approx 260\,\mathrm{mK}$, supporting the plausible conclusion that this phenomenon is intrinsic to herbertsmithite single crystals in ambient magnetic fields of ${|B|}\le 5\,{\rm{\mu }}{\rm{T}}.$

### Spinon-mediated interactions via *Z*_2_ QSL

The Hamiltonian for mutual witness spin interactions in equations (1) and (2)^40,64^ is derived from the coupling between a witness spin ${{\boldsymbol{s}}}_{i}$ and a kagome spin ${{\boldsymbol{s}}}_{l}^{{\rm{Kagome}}}$ as

9 $${H}_{{\rm{coupling}}}=\gamma {{\boldsymbol{s}}}_{i}\cdot {{\boldsymbol{s}}}_{l}^{{\rm{Kagome}}}.$$

The intra-kagome spin susceptibility ${\zeta }_{{lm}}$ is calculated using linear response equations (3) and (4) from the spinon band structure of a Z_2_$\left[0,\,{\rm{\pi }}\right]\beta$ QSL. A Z_2_$\left[0,\,{\rm{\pi }}\right]\beta$ QSL is the only gapped QSL that is compatible with lattice symmetries at the mean field level, and is in the neighbourhood of the U(1)$\left[0,\,{\rm{\pi }}\right]$ state whose energy is the lowest among different U(1) QSLs^28^.

In units of the nearest-neighbour spinon hopping energy ${t}_{1}=0.4{J}_{{\rm{K}}}\approx 76\,{\rm{K}}$ (ref. ^28^), the Z_2_$\left[0,\,{\rm{\pi }}\right]\beta$ QSL Hamiltonian contains real parameters for the second-neighbour hopping ${t}_{2}$, gap ${\Delta }_{2}$, and two Lagrange multipliers ${\lambda }_{1}$ and ${\lambda }_{3}$ which enforce the physical Hilbert space constraint of half-filling. We solve for all four using the standard self-consistent mean field approach. Reference ^36^ gives the following mean field spinon Hamiltonian for the kagome planes:

10 $${{\boldsymbol{s}}}_{l}^{\mathrm{Kagome}}=\frac{1}{2}\mathop{\sum }\limits_{\alpha ,\beta =\left\{\uparrow ,\downarrow \right\}}{f}_{i\alpha }^{\,\dagger }{\sigma }_{\alpha \beta }\,{f}_{i\beta },$$

11 $$\begin{array}{l}{\hat{H}}_{\mathrm{QSL}}=\left\{{\mathop{\sum}\limits_{i}^{N}}{\lambda }_{3}\left({f}_{i\uparrow }^{\,\dagger }{f}_{i\uparrow }+{f}_{i\downarrow }^{\,\dagger }{f}_{i\downarrow }\right)+{\lambda }_{1}\left({f}_{i\uparrow }^{\,\dagger }{f}_{i\downarrow }^{\,\dagger }+{f}_{i\downarrow }{f}_{i\uparrow }\right)\right\}\\\qquad\quad+\left\{\mathop{\sum }\limits_{ij}\left({t}_{1}{\nu }_{ij}^{\left(1\right)}+{t}_{2}{\nu }_{ij}^{\left(2\right)}\right)\left({f}_{i\uparrow }^{\,\dagger }{f}_{j\uparrow }+{f}_{i\downarrow }^{\,\dagger }{f}_{j\downarrow }-{f}_{i\uparrow }{f}_{j\uparrow }^{\,\dagger }-{f}_{i\downarrow }{f}_{j\downarrow }^{\,\dagger }\right)\right.\\\left.\qquad\quad\qquad\quad+{\Delta }_{2}{\nu }_{ij}^{\left(2\right)}\left({f}_{i\uparrow }^{\,\dagger }{f}_{j\downarrow }^{\,\dagger }-{f}_{i\downarrow }^{\,\dagger }{f}_{j\uparrow }^{\,\dagger }-{f}_{i\uparrow }{f}_{j\downarrow }+{f}_{i\downarrow }\,{f}_{j\uparrow }\right)\vphantom{\mathop{\sum }\limits_{ij}}\right\},\end{array}$$

where ${\nu }_{{ij}}^{\left(1\right)}$ is non-zero only for first-nearest neighbours (and is 1 or −1 as defined in ref. ^36^), and ${\nu }_{{ij}}^{\left(2\right)}$ is non-zero only for second-nearest neighbours. There are N sites in the system. It is convenient to rewrite the diagonal terms using

12 $$\left\{{f}_{i},{f}_{j}^{\,\dagger }\right\}={\delta }_{{ij}},$$

13 $$\left\{{f}_{i},{f}_{j}\right\}=0,$$

which gives

14 $$\begin{array}{l}{\hat{H}}_{\mathrm{QSL}}=N{\lambda }_{3}+\left\{\mathop{\sum }\limits_{i}\frac{{\lambda }_{3}}{2}\left({f}_{i\uparrow }^{\,\dagger }{f}_{i\uparrow }-{f}_{i\uparrow }{f}_{i\uparrow }^{\,\dagger }+{f}_{i\downarrow }^{\,\dagger }{f}_{i\downarrow }-{f}_{i\downarrow }{f}_{i\downarrow }^{\,\dagger }\right)\right.\\\left.\qquad\qquad\qquad\qquad\quad\;+\frac{{\lambda }_{1}}{2}\left({f}_{i\uparrow }^{\,\dagger }{f}_{i\downarrow }^{\,\dagger }+{f}_{i\downarrow }{f}_{i\uparrow }-{f}_{i\downarrow }^{\,\dagger }{f}_{i\uparrow }^{\,\dagger }-{f}_{i\uparrow }{f}_{i\downarrow }\right)\vphantom{\mathop{\sum }\limits_{i}}\right\}\\\qquad\qquad\quad\quad\;+\left\{\mathop{\sum }\limits_{ij}\left({t}_{1}{\nu }_{ij}^{\left(1\right)}+{t}_{2}{\nu }_{ij}^{\left(2\right)}\right)\left({f}_{i\uparrow }^{\,\dagger }{f}_{j\uparrow }+{f}_{i\downarrow }^{\,\dagger }{f}_{j\downarrow }-{f}_{i\uparrow }{f}_{j\uparrow }^{\,\dagger }-{f}_{i\downarrow }{f}_{j\downarrow }^{\,\dagger }\right)\right.\\\left.\qquad\qquad\qquad\qquad\quad\;+{\Delta }_{2}{\nu }_{ij}^{\left(2\right)}\left({f}_{i\uparrow }^{\,\dagger }{f}_{j\downarrow }^{\,\dagger }-{f}_{i\downarrow }^{\,\dagger }{f}_{j\uparrow }^{\,\dagger }-{f}_{i\uparrow }{f}_{j\downarrow }+{f}_{i\downarrow }{f}_{j\uparrow }\right)\vphantom{\mathop{\sum }\limits_{ij}}\right\}.\end{array}$$

The initial $N{\lambda }_{3}$ acts as an overall chemical potential and can be dropped. The following basis is then block diagonal:

15 $${\hat{H}}_{\mathrm{QSL}}=\mathop{\sum }\limits_{ij}\left(\begin{array}{cc}\left(\begin{array}{cc}{f}_{i\uparrow }^{\,\dagger } & {f}_{i\downarrow }\end{array}\right) & \left(\begin{array}{cc}{f}_{i\uparrow } & {f}_{i\downarrow }^{\,\dagger }\end{array}\right)\end{array}\right)\left(\begin{array}{cc}({h}_{{ij}}) & (0)\\ (0) & (-{h}_{{ij}})\end{array}\right)\left(\begin{array}{l}\left(\begin{array}{l}{f}_{j\uparrow }\\ {f}_{j\downarrow }^{\,\dagger }\end{array}\right)\\ \left(\begin{array}{l}{f}_{j\uparrow }^{\,\dagger }\\ {f}_{j\downarrow }\end{array}\right)\end{array}\right),$$

where

16 $${h}_{{ij}}=\left(\begin{array}{cc}\frac{{\lambda }_{3}}{2}{\delta }_{{ij}}+{t}_{a}{\nu }_{{ij}}^{a} & \frac{{\lambda }_{1}}{2}{\delta }_{{ij}}+{\Delta }_{2}{\nu }_{{ij}}^{\left(2\right)}\\ \frac{{\lambda }_{1}}{2}{\delta }_{{ij}}+{\Delta }_{2}{\nu }_{{ij}}^{\left(2\right)} & -\frac{{\lambda }_{3}}{2}{\delta }_{{ij}}-{t}_{a}{\nu }_{{ij}}^{a}\end{array}\right)$$

(a sum over a=1,2 is implicit). Hence, all the information is contained in the upper matrix:

17 $${\hat{H}}_{\mathrm{QSL}}^{U}=\mathop{\sum }\limits_{ij}{\left(\begin{array}{cc}{f}_{i\uparrow }^{\,\dagger } & {f}_{i\downarrow }\end{array}\right)}_{\alpha }{h}_{{ij}}^{\alpha \beta }{\left(\begin{array}{l}{f}_{j\uparrow }\\ {f}_{j\downarrow }^{\,\dagger }\end{array}\right)}_{\beta }.$$

The self-consistency conditions are

18 $${\Delta }_{{ij}}=-2\left\langle {f}_{i\uparrow }{f}_{j\downarrow }\right\rangle =2\left\langle {f}_{i\downarrow }\,{f}_{j\uparrow }\right\rangle ,$$

19 $${t}_{{ij}}=2\left\langle {f}_{i\uparrow }^{\,\dagger }{f}_{j\uparrow }\right\rangle =2\left\langle {f}_{i\downarrow }^{\,\dagger }{f}_{j\downarrow }\right\rangle ,$$

20 $$0=\left\langle {f}_{i\uparrow }{f}_{j\uparrow }\right\rangle =\left\langle {f}_{i\downarrow }{f}_{j\downarrow }\right\rangle =\left\langle {f}_{i\uparrow }^{\,\dagger }{f}_{j\downarrow }\right\rangle =\left\langle {f}_{i\downarrow }^{\,\dagger }{f}_{j\uparrow }\right\rangle .$$

The global half-filling constraint on the physical Hilbert space is enforced by the Lagrange multipliers ${\lambda }_{1}$ and ${\lambda }_{3}$:

21 $${\lambda }_{1}:0=\mathop{\sum }\limits_{i}\left\langle {f}_{i\uparrow }{f}_{i\downarrow }\right\rangle -\left\langle {f}_{i\downarrow }{f}_{i\uparrow }\right\rangle ,$$

22 $${\lambda }_{3}:1=\mathop{\sum }\limits_{i}\left\langle {f}_{i\uparrow }^{\,\dagger }{f}_{i\uparrow }\right\rangle +\left\langle {f}_{i\downarrow }^{\,\dagger }{f}_{i\downarrow }\right\rangle .$$

This must now be diagonalized:

23 $${\hat{H}}_{\mathrm{QSL}}^{U}=\mathop{\sum }\limits_{ij}\left(\begin{array}{cc}{\gamma }_{i1}^{\dagger } & {\gamma }_{i2}^{\dagger }\end{array}\right){D}_{{ij}}\left(\begin{array}{l}{\gamma }_{j1}\\ {\gamma }_{j2}\end{array}\right)$$

with diagonal D, and

24 $$h={UD}{U}^{\dagger },$$

25 $$\left(\begin{array}{c}{f}_{i\uparrow }\\ {f}_{i\downarrow }^{\dagger }\end{array}\right)={U}_{{ij}}\left(\begin{array}{c}{\gamma }_{j1}\\ {\gamma }_{j2}\end{array}\right)=\left(\begin{array}{c}{U}_{{ij}}^{11}{\gamma }_{j1}+{U}_{{ij}}^{12}{\gamma }_{j2}\\ {U}_{{ij}}^{21}{\gamma }_{j1}+{U}_{{ij}}^{22}{\gamma }_{j2}\end{array}\right),$$

and the Hermitian conjugate gives the other required terms:

26 $$\left(\begin{array}{cc}{f}_{i\uparrow }^{\,\dagger } & {f}_{i\downarrow }\end{array}\right)=\left(\begin{array}{cc}{U}_{{ij}}^{11* }{\gamma }_{j1}^{\dagger }+{U}_{{ij}}^{12* }{\gamma }_{j2}^{\dagger } & {U}_{{ij}}^{21* }{\gamma }_{j1}^{\dagger }+{U}_{{ij}}^{22* }{\gamma }_{j2}^{\dagger }\end{array}\right).$$

In this basis,

27 $$\left\{{\gamma }_{i\alpha },{\gamma }_{j\beta }\right\}=\left\{{\gamma }_{i\alpha }^{\dagger },{\gamma }_{j\beta }^{\dagger }\right\}=0,$$

28 $$\left\{{\gamma }_{i\alpha },{\gamma }_{j\beta }^{\dagger }\right\}={\delta }_{{ij}}{\delta }_{\alpha \beta },$$

29 $$\left\langle {\gamma }_{i\alpha }^{\dagger }{\gamma }_{j\beta }\right\rangle ={\delta }_{{ij}}{\delta }_{\alpha \beta }{n}_{{\rm{D}}}\left({D}_{{ii}}^{\alpha \alpha }\right),$$

where ${n}_{{\rm{D}}}$ is the Fermi–Dirac distribution. However, note that the eigenvalues ${D}_{{ii}}$ are ordered low to high, and the spectrum is symmetric about zero. Hence, at T≈0 (since ${T}^{* }/{J}_{{\rm{K}}}=0.26\,{\rm{K}}/190\,{\rm{K}}\ll 1$), ${n}_{{\rm{D}}}\left({D}_{{mm}}^{22}\right)=0$ and ${n}_{{\rm{D}}}\left({D}_{{mm}}^{11}\right)=1$. Feeding these expressions into the self-consistency conditions gives

30 $${\Delta }_{2}{\nu }_{{ij}}^{\left(2\right)}=2\mathop{\sum }\limits_{{{m}}}{U}_{{im}}^{11}{U}_{{jm}}^{21* },$$

31 $${t}_{a}{\nu }_{{ij}}^{\left(a\right)}=2\mathop{\sum }\limits_{{{m}}}{U}_{{im}}^{11* }{U}_{{jm}}^{11},$$

32 $${\lambda }_{1}:0=\mathop{\sum }\limits_{im}{U}_{{im}}^{12}{U}_{{im}}^{22* }-{U}_{{im}}^{21* }{U}_{{im}}^{11},$$

33 $${\lambda }_{3}:1=\mathop{\sum }\limits_{i}{\left|{U}_{{ii}}^{11}\right|}^{2}+{\left|{U}_{{ii}}^{22}\right|}^{2}.$$

We set ${t}_{1}=1$, defining the energy scale. Working in q space at ${q}=\,0$ (since the gap should be constant), we found a self-consistent solution with

34 $${\Delta }_{2}=0.4583,$$

35 $${t}_{2}=-0.2849,$$

36 $${\lambda }_{1}=0.4327,$$

37 $${\lambda }_{3}=1.500.$$

We used a tolerance of ${10}^{-3}$ in finding the constraints with the Lagrange multipliers:

38 $${\lambda }_{1}\Rightarrow \mathop{\sum }\limits_{im}{U}_{{im}}^{12}{U}_{{im}}^{22* }-{U}_{{im}}^{21* }{U}_{{im}}^{11}=-8.4\times {10}^{-4}\,\left( \sim 0\right),$$

39 $${\lambda }_{3}\Rightarrow \mathop{\sum }\limits_{i}{|{U}_{{ii}}^{11}|}^{2}+{|{U}_{{ii}}^{22}|}^{2}=1.001\left( \sim 1\right).$$

The overall energy gap (identified from the density of states),

40 $$2\Delta =0.44{t}_{1}=33\,{\rm{K}},$$

is essentially equal to the gap ($0.43{t}_{1}$) identified previously using exact diagonalization^39^.

In the witness–witness spin interactions ${J}_{{ij}}$ in equations (1)–(4), the only free parameter is γ. We constrain $|\gamma |=60\,{\rm{K}}\approx {J}_{{\rm{K}}}/3$ by requiring a match to the widely reported experimental value of the Curie–Weiss temperature ${\theta }_{{\rm{CW}}}\left(1\,{\rm{K}} < T\right)=-1.1\,{\rm{K}}$.

One plausibly estimates^41,42^

41 $${r}_{0}=\frac{\hslash {v}_{{\rm{F}}}}{2\Delta },$$

where the spinon band structure enters via the gap 2Δ, and the spinon Fermi velocity ${v}_{{\rm{F}}}$ of the parent U(1)$\left[0,\,{\rm{\pi }}\right]$ gapless QSL from which the Z_2_$\left[0,\,{\rm{\pi }}\right]\beta$ forms^28^ with

42 $${v}_{{\rm{F}}}=\frac{{\sqrt{2}t}_{1}d}{\hslash },$$

where d is the nearest-neighbour kagome Cu spacing. Equations (41) and (42) lead to equation (6).

### Witness spin Monte Carlo simulations

In herbertsmithite, the witness spin sites (that is, Zn^2+^ sites) form a triangular lattice on the ab plane, staggered along the c axis with a period of 3. This witness spin lattice effectively connects as a simple cubic lattice^17^. We simulate a witness spin lattice with the size of $X\times X\times Z=45\times 45\times 4.$ The smallest cell containing one witness spin, which is $1\times 1\times 1$ under this notation, is a rhombic prism with the side length $a/\sqrt{3}=3.95\,\mathring{\rm A}$ and height $c=14.09\,\mathring{\rm A}$. The direction of its rhombic base is rotated by 90° around the *c* axis, compared with the rhombic base of the conventional unit cell of herbertsmithite. To satisfy periodic boundary conditions, X has to be a multiple of three and Z has to be an even number.

We created a Monte Carlo simulation of the witness spins using the Metropolis–Hasting algorithm. We modelled the witness spins as classical Ising spins ${s}_{i}=\pm 1/2$. Although witness spins in herbertsmithite are not Ising like, they are not Heisenberg like, either. Electron spin resonance demonstrates a strong Dzyaloshinskii–Moriya interaction ($D/{J}=\,0.08$), leading to spin anisotropy^45^. Moreover, there is evidence for additional easy-axis anisotropy beyond the Dzyaloshinskii–Moriya interaction^65^. A key consequence of this magnetic anisotropy is that using a pure Heisenberg representation to model witness spin dynamics would be incorrect and that using an Ising representation is a reasonable approximation choice. The use of Ising spins has a further pragmatic justification in the context of spin glass. Adding a tiny amount of anisotropy to a Heisenberg spin glass can lead to a spin glass in the Ising universality class, making the Ising-spin model effective in reproducing experimental observations^66^. We initialized the system with the size 45 × 45 × 4 and periodic boundary conditions with 33% of potential witness spin sites occupied (N=2,673 spins). The configuration of occupied sites is randomly assigned using a seeded random number generator. We average all of our results over 128 different configurations, keeping the same sets of seed across all runs. For each seeded configuration, we also average our results over three simulation runs.

In all cases, the initial state of the system has each spin in random uncorrelated states, corresponding to infinite temperature. We then run our simulation starting at ${T}=\,$ 400 mK—above the freezing transition temperature so as to avoid quenching the glass—and ending at 50 mK at intervals of 10 mK. In addition, we also conduct a separate run going from 10 K to 1.6 K at intervals of 0.2 K to ensure that the Curie–Weiss temperature is ${\theta }_{{\rm{CW}}}=-1.1\,$ K, and from 1.5 K to 0.5 K at intervals of 0.1 K for completeness.

At each temperature point, we first equilibrate the system by updating the system over 1,000 sweeps, with each sweep consisting of N=2,673 update steps. We then sampled the spin-per-site noise $s\left(t\right)=\frac{1}{N}{\sum }_{i}{s}_{i}(t)$, EA spin glass order parameter ${q}_{{\rm{EA}}}=\frac{1}{N}{\sum }_{i}{\overline{2{s}_{i}\left(t\right)}}^{\,2}$ and AF order parameter ${\phi }_{\mathrm{AF}}=\overline{\frac{1}{N}{\left({\sum }_{i}{\left(-1\right)}^{k}(2{s}_{i}(t))\right)}^{2}}$ ($k=\mathrm{0,1}$ for each sublattice of the bipartite witness spin sites) over 100,000 sweeps. The bar represents an average over the Monte Carlo sweep time. From $s\left(t\right)$, the magnetization noise ${{M}}(t)$ and d.c. magnetic susceptibility χ are estimated using

43 $${{M}}\left(t\right)={\rho }_{V}{\mu }_{0}g{\mu }_{{\rm{B}}}s\left(t\right)\sqrt{\frac{N}{{N}_{{\rm{EXP}}}}},$$

44 $$\chi ={\rho }_{V}{\mu }_{0}{\left(g{\mu }_{{\rm{B}}}\right)}^{2}N\frac{\overline{{s}^{2}\left(t\right)}-{\left(\overline{s\left(t\right)}\right)}^{2}}{{k}_{{\rm{B}}}T},$$

where ${\rho }_{V},{\mu }_{0},g\,=\,2,\,{\mu }_{{\rm{B}}}\,\mathrm{and}\,{k}_{{\rm{B}}}$ are the number density per volume of witness spins in herbertsmithite (33% per Zn sites), vacuum permeability, electron g-factor, Bohr magneton and Boltzmann constant, respectively. The factor $\sqrt{N/{N}_{{\rm{EXP}}}}$, where ${N}_{{\rm{EXP}}}$ is the number of witness spins in the volume of herbertsmithite Sample 1 (~3 mm^3^), is required to approximately estimate the order of the magnetization noise magnitude that generally scales as ${{M}}(t)\propto 1/\sqrt{{N}_{{\rm{EXP}}}}$ (ref. ^32^). The error bars of χ, ${\phi }_{{\rm{AF}}}$ and ${q}_{{\rm{EA}}}$ are the standard error of averaging.

The predicted witness spin magnetization noise ${{M}}(t)$ is then processed in the same method as the experimental spin noise in ZnCu_3_(OH)_6_Cl_2_ (see the ‘Measurements’ section). The distribution is Gaussian with small statistical fluctuations (Extended Data Fig. 4a). The PSD in Fig. 4c,d and Extended Data Fig. 4b has a frequency resolution of $\Delta \omega =\left(2{\rm{\pi }}\,{\rm{rad}}\right)\times \left({10}^{-5}/{\rm{MCS}}\right)=6\times {10}^{-5}$ rad/MCS and is averaged over a 10$\Delta \omega$ or $100\Delta \omega$ window at high frequencies; the error bars are the standard error of averaging. The power index in Fig. 5a is obtained by fitting in $6\times {10}^{-5}$ rad/MCS $\le \omega \le$ $1\times {10}^{-3}$ rad/MCS (Extended Data Fig. 4b); the error bars are the standard error from fitting. In Fig. 5a, open symbol points are used at temperatures above ${T}^{* }$ at which the power-law fitting is challenging (*R* < 0.98). The variance in Fig. 5b is calculated by integrating the PSD from $6\times {10}^{-5}$ rad/MCS $\le \omega \le$ $6\times {10}^{-2}$ rad/MCS. The variance peak temperature remains at ${T}^{* }$ for different integration ranges (Extended Data Fig. 4c). When 1 MCS = 100 μs, the simulated PSD and the measured experimental PSD roughly correspond in the same frequency window (Extended Data Fig. 5). They remain consistent with each other for any values in the range of 1 MCS < 100 μs, as long as the PSD continues to be scale invariant down to lower frequency in both simulation and experiment. The challenges in fitting PSD at temperatures above ${T}^{* }$, which were not seen in the experiment, may be resolved by simulating the PSD in a lower Monte Carlo frequency range or by simulating the fully quantum mechanical theory.

When we perform the equivalent simulations for different witness spin concentrations from 15% to 60%, the transition temperature ${T}^{* }$ changes from 200 mK to 100 mK, and the nearest-neighbour witness spin interaction energy scale (equation (7)) changes from 2.5 K to 0.5 K. Even if these different witness spin concentrations are used in the model, these quantitative changes do not alter the conclusion of this work.

We finally note that whether the predicted transition is of true spin glass type has not been examined in detail. Direct theoretical investigations into this point, such as finite-size scaling of the spin glass susceptibility, are left for future work.

### Neutron scattering structure factor

Extended Data Fig. 6 shows the witness spin structure factor ${\Sigma} ({\boldsymbol{q}})$ that is calculated from a spin configuration snapshot in our Monte Carlo simulation at 2 K over 1,000 sweeps and averaging over 128 configurations:

45 $${\Sigma} \left({\boldsymbol{q}}\right)=\left|F\left({\boldsymbol{q}}\right)\right|^{2}\,\left\langle \left|\sum \limits_{i}{s}_{i}{{\rm{e}}}^{{{i}}{\boldsymbol{q}}\cdot {{\boldsymbol{r}}}_{i}}\right|^{2}\right\rangle ,$$

where $F({\boldsymbol{q}})$ is the magnetic form factor of Cu^2+^. It reasonably matches the low-energy neutron scattering structure factor in ZnCu_3_(OH)_6_Cl_2_, which shows diffuse scattering without a sharp peak^17,26^, although with a fairly low signal-to-noise ratio. Comparable features are the shape of the mid-intensity contribution (green) that extends throughout the in-plane and out-of-plane directions, the high-intensity contribution (red) at the out-of-plane peak at (00$\frac{3}{2}$), and the high-intensity in-plane circular shape contribution with correct $|{\boldsymbol{q}}|$ and approximately equally distributed intensity. Further comparison of the precise shape of the experimental neutron scattering intensity pattern in the (*HK*0) plane requires improvement both in the precision of the neutron scattering experiment and in the model.

### Considering alternatives to spinon-mediated witness spin interactions

Although we considered a large number of alternative hypotheses, the only cases capable of explaining the full range of experimental data involved spinon-mediated couplings via spin liquids. In reviewing these hypotheses, there are two strong constraints.

First, any theoretical model with sufficiently rapid variation with distance of the witness spin to witness spin interaction decay (local couplings) will result in a sizable population of isolated witness spins. With Zn site occupation probability p, the percentage of isolated witness spins having no nearest neighbour is ${\left(1-p\right)}^{6}$; with p=0.33, this gives 9% (3% of Zn sites). These isolated witness spins must contribute a d.c. magnetic susceptibility, which diverges as 1/T as T→0. As shown in Extended Data Fig. 7a, if only 0.7% of Zn sites are occupied by isolated witness spins, this would be enough to show a d.c. susceptibility evolving as 1/T as T→0, which is not observed in any of our experiments.

Second, witness spin to witness spin interactions evolving too slowly with distance ($1/{r}^{2}$ or slower) will lead to an unphysical divergence in the sum over spins forming the structure factor. As well as being unphysical, this situation is incompatible with the experimental inelastic neutron scattering structure factor that shows broad features in momentum space at 2 K, consistent with dominant AF nearest-neighbour correlations^17^. An example of the structure factor for a slowly decaying spin-wave mediated interaction is shown in Extended Data Fig. 7b,c.

In the context of these constraints, we have considered and ruled out a variety of alternatives to spinon-mediated witness spin interactions including nearest-neighbour local exchange, next-nearest-neighbour local exchange, direct dipolar witness spin interactions, dimer correlations mediating the witness spin interaction, random singlets and random spin clusters, and spin-wave mediating the witness spin interaction (Supplementary Discussion).

The model in the main text discusses the Z_2_$\left[0,\,{\rm{\pi }}\right]\beta$ QSL scenario, whereas another candidate kagome QSL in herbertsmithite is the U(1)[0, ${\rm{\pi }}$] state with a Dirac nodal spinon Fermi surface. We calculated its spinon band structure using the mean field decoupling of ref. ^28^ and then the witness spin interaction using the same methods we used for Z_2_: in this case, the calculation becomes that of an Ruderman-Kittel-Kasuya-Yosida interaction between witness spins mediated by the spinon Dirac nodal Fermi surface. We find that all witness couplings are AF, decaying approximately as $1/{r}^{3}$. The predictions of the witness spin noise, susceptibility and order parameters via these U(1) QSL spinon-mediated witness spin interactions are shown in Extended Data Fig. 8. Each panel can be compared with Figs. 4 and 5. The U(1) model predicts a transition at 110 mK, which is smaller than the value of 150 mK of the Z_2_ QSL prediction. Thus, our Z_2_ QSL model is more consistent with the experiment. However, the U(1) QSL model also reproduces the qualitative features of the experiment in Figs. 2 and 3, and cannot be fully excluded using the existing data.

## Online content

Any methods, additional references, Nature Portfolio reporting summaries, source data, extended data, supplementary information, acknowledgements, peer review information; details of author contributions and competing interests; and statements of data and code availability are available at 10.1038/s41567-026-03303-6.

## Supplementary information

Supplementary Information Supplementary Discussion.

## Source data

Source Data Fig. 2 Statistical source data.

Source Data Fig. 3 Statistical source data.

Source Data Fig. 4 Statistical source data.

Source Data Fig. 5 Statistical source data.

Source Data Extended Data Fig./Table 1 Statistical source data.

Source Data Extended Data Fig./Table 2 Statistical source data.

Source Data Extended Data Fig./Table 3 Statistical source data.

Source Data Extended Data Fig./Table 4 Statistical source data.

Source Data Extended Data Fig./Table 5 Statistical source data.

Source Data Extended Data Fig./Table 6 Statistical source data.

Source Data Extended Data Fig./Table 7 Statistical source data.

Source Data Extended Data Fig./Table 8 Statistical source data.

## Data Availability

The data presented in this paper are available via Zenodo at 10.5281/zenodo.15114443 (ref. ^67^). Source data are provided with this paper.

## Code availability

The simulation codes used in this paper are available via Zenodo at 10.5281/zenodo.15114443 (ref. ^67^).
