# Supplementary material for: Spinon mediation of witness spin dynamics in herbertsmithite
Source: Nat Phys. 2026 Jun 10;22(7):1071–8. doi: 10.1038/s41567-026-03303-6 (PMC13423850; doi:10.1038/s41567-026-03303-6)
Supplement: Supplementary file 1 — Supplementary Discussion. [file 41567_2026_3303_MOESM1_ESM.pdf]

nature physics

Supplementary information

https://doi.org/10.1038/s41567-026-03303-6

Spinon mediation of witness spin dynamics in herbertsmithite

In the format provided by the authors and unedited

Supplementary Discussion

A. Hypothetical alternatives to spinon-mediated witness spin interactions

1. *Nearest neighbor (NN) local exchange*

Antiferromagnetic (AF) NN witness spin interactions could occur via some sequence of exchange pathways[17]. The shortest path would be Cu-O-O-Cu (super-super exchange) connecting two witness planes. However, this NN case is un-frustrated for either ferromagnetic (FM) or AF interactions, and so cannot account for the observed spin glass transition at T^*. Models of random NN exchange with a Gaussian distribution of values centered on the net AF interaction strength (1 K) seen in neutron scattering, give rise to long-range AF order; we confirmed this by simulation. Finally, any NN model would also leave ~9% of isolated witness spins in our samples, inconsistent with the observed DC magnetic susceptibility as $T \to 0$.

2. *Next-nearest neighbor (NNN) local exchange*

In principle, some as-yet unknown AF (frustrating) NNN exchange pathway might exist. However, there is no evidence of NNN correlations in neutron scattering[17]. Even if NNN interactions exist and are captured by an AF NN+NNN model, NN interactions would give FM order on triangular witness spin planes that are then AF coupled from plane to plane, and NNNs would then frustrate the in-plane FM order. The resulting order would likely be the standard 120-degree order on a witness spin plane which is AF coupled from plane to plane. To explore this concept we modeled a range of AF NNN interaction strengths from 0 to the NN strength in our MC simulations. We found that all simulations led to sizable AFM order parameters $\phi_{\mathrm{AF}} > 0.4$ (cf. $\phi_{\mathrm{AF}} < 0.02$ in Fig. 5d for the Z_2 predicted QSL model) suggesting significant long-range order, which is not detected in specific heat or neutron scattering measurements. Finally, any NN+NNN model would also leave ~2.4% of isolated witness spins, again inconsistent with the observed DC magnetic susceptibility as $T \to 0$.

3. *Direct dipolar witness spin interactions*

While long-range dipolar witness spin interactions could be frustrating, the associated energy scale is too small. With a NN witness-witness distance of 6.1 Å, the NN dipole-dipole interaction energy between two $s = 1/2$ spins is

$$\frac{J_{\mathrm{NN}}}{k_{\mathrm{B}}} = \frac{1}{k_{\mathrm{B}}}\frac{\mu_0}{4\pi}\frac{(2\mu_{\mathrm{B}})^2}{\left(6.1\ \text{Å}\right)^3} = 11\ \mathrm{mK}, \tag{46}$$

which is incompatible with the observed $T^* = 260\ \mathrm{mK}$. Since NN interactions are unfrustrated, any predicted T^* would rely on further neighbor couplings and so would be reduced even further below this estimated temperature.

4. *Dimer correlations mediating the witness spin interaction*

One can hypothesize effects of Zn substituted into the Cu Kagome plane[11,19,23,48,55]. Ref. 68 suggests that two spins on each triangle containing a Zn-substituted site may form an un-frustrated singlet (dimer). However, for such a hypothesis ref. 69 calculates the Knight shift around Zn-substitution and finds it to decay as $1/r$; such a slow decay is inconsistent with the experimental structure factor. Further, ref. 48 suggests that these disturbances lead to a Curie-like $1/T$ susceptibility, incompatible with the the observed DC magnetic susceptibility as $T \to 0$.

There is also a theoretical suggestion that a free-spin contribution might arise in the kagome layers due to Zn-substitution[70]. However, such a contribution is inconsistent with both the experimental structure factor which indicates that the witness spins are embedded in the Zn layers[17], and with the observed DC magnetic susceptibility as $T \to 0$.

5. *Random singlets and random spin clusters*

Power-law scaling in herbertsmithite has been identified in specific heat[52,71] and the dynamic spin susceptibility[25], which led to the hypothesis of a random singlet state arising from randomly distributed AF interactions. Ref. 72 studies random singlets and predicts, as a function of decreasing temperature, a magnetic susceptibility that increases as $1/T$, then plateaus, then increases as $1/T^a$ with $a < 1$. A recent study on an organic QSL candidate

similarly found a low-T upturn related to random singlets[73]. However, herbertsmithite shows no susceptibility divergence in the observed DC magnetic susceptibility as $T \rightarrow 0$.

Ref. 72 also discusses qualitatively that coexisting random FM & AF interactions could lead to a partial formation of FM spin clusters of random size. However, there is no evidence of FM correlations in the neutron scattering structure factor. Also, the derived witness spin interactions mediated by Z_2 or U(1) quantum spin liquids (see below) are purely AF, and therefore are incompatible with a FM cluster formation scenario.

6. *Spin-wave mediating the witness spin interaction*

Spin-wave-like excitations could be seen in an insulating spin glass, for example in $Eu_xSr_{1-x}S$ [74], and the method detailed in ref. 40 for spin-wave mediated nuclear spin coupling in herbertsmithite could potentially also apply to witness spin coupling. To account for this picture, we modelled spin-wave-mediated witness spin interactions using the Kondo-Yamaji Green's function decoupling of ref. 40. The interaction oscillates in sign and decays as $1/r^2$, however, it decays as $1/r$ along high-symmetry directions. Such a slow decay is inconsistent with the experimental structure factor, as shown in Extended Data Figs. 7b,c.

Furthermore, spin waves or magnons have never been identified in herbertsmithite, even by inelastic neutron scattering at 50 mK[17,26,27], thus excluding a spin-wave mediation scenario.

B. General Impurity Spin Effects

1. Role of impurities in herbertsmithite and possible interaction with quantum spin liquids

The quasi-free "impurity" spin contribution has been known to exist in herbertsmithite since the pioneering studies[12,13,14,19,55], and there has been discussions on how it could affect the physics of herbertsmithite.

While impurities and kagome spins tended to be treated as a separate contribution in experimental analyses, the interplay between them gradually became regarded as relevant. To list a few examples, there has been discussions on a possible coupling of impurity spins to spinons to form a Kondo type ground state[22,28], a possible Jahn–Teller driven distortion

leading to a displacement of the six adjacent oxygens to induce an in-plane staggered magnetic response[23], and impurity-scattering of thermal carriers[53].

Ref. 23 points out that impurity spins could offer a handle to probe the physics of the kagome layer. Our work concretely formulates and executes this concept that spin noise dynamics of "impurity" witness spins can probe the kagome layer physics, and quantitatively demonstrates that this approach is successful in deducing spinon-mediated interaction mechanisms via the kagome quantum spin liquid.

2. Spin glass transitions in spin liquid candidates

There are several materials classes that are hypothesized to be spin liquids yet exhibit a spin-glass transition at low temperatures. Here we list an example from each of Cu, Yb, Ir, Cr, Fe, and Gd-based compound families.

- $Cu_3V_2O_7(OH)_2\cdot 2H_2O$: Cu^{2+} ions with $S = 1/2$ form a distorted kagome lattice in a monoclinic structure. It exhibits a spin freezing at 1.2 K [75], or at 60 mK in another sample[56], despite Curie-Weiss temperature of $\theta_{\mathrm{CW}} = -115$ K. The transition is proposed to arise from freezing of defect-induced moments within the kagome plane[56].
- $YbMgGaO_4$: Yb^{3+} (spin-orbital $J_{\mathrm{eff}} = 1/2$) form a triangular lattice, with site-mixing disorder of non-magnetic Mg^{2+} and Ga^{3+} causing random local Yb^{3+}-environment distortion[76]. It exhibits a spin freezing at ~ 0.1 K (θ_{CW} of -10 K order), suggested to be driven by disorder and frustration[77].
- $Na_4Ir_3O_8$: Ir^{4+} (spin-orbital $J_{\mathrm{eff}} = 1/2$) form a hyperkagome lattice, exhibiting a disordered magnetic freezing of all Ir^{4+} sites at ~ 7 K [78] ($\theta_{\mathrm{CW}} \sim -650$ K [79]). The inhomogeneous magnetic ground state is proposed to be driven either by disorder inherent to the creation of the hyperkagome lattice or via quantum fluctuations[80].
- $SrCr_{9p}Ga_{12\text{-}9p}O_{19}$: Cr^{3+} ($S = 3/2$) form a kagome-triangular-kagome trilayer lattice[81]. It shows a spin-glass-like transition at $\sim$3 K ($\theta_{\mathrm{CW}} \sim -500$ K) in a wide range of stoichiometry $p > 0.6$, which occurs in bulk but not due to isolated impurities[82]. The transition has been given different interpretations, for example, freezing of magnetic defects localized around spin vacancies[82], or a spin jam state caused by quantum fluctuations in a disorder-free lattice[83].

• $(H_3O)Fe_3(SO_4)_2(OH)_6$: Fe^{3+} ($S = 5/2$) form a kagome lattice, showing a spin glass transition at 17 K ($\theta_{\mathrm{CW}} \sim -1200$ K)[84]. The transition is proposed to be due to a coherent anisotropic distortion of oxygen octahedra that generates in-plane anisotropy, not from random disorder[85].

• $Gd_3Ga_5O_{12}$: Gd^{3+} ($S = 7/2$) form a hyperkagome lattice, showing a spin glass transition at 0.14 K ($\theta_{\mathrm{CW}} \sim -2$ K) [63]. The transition is proposed to be due to highly frustrated geometry of the magnetic lattice[63], or a mixture of a spin-liquid state with a set of rigid magnetic pieces nucleated around impurity centers[86].

Importantly, for all these QSL candidates, the microscopic spin physics is quite different from that of herbertsmithite. The majority of these compounds have spin $S \geq 3/2$ or spin-orbital $J_{\mathrm{eff}} = 1/2$ each of which are quantum-mechanically distinct nature from pure $S = 1/2$ in herbertsmithite. Further, it is the spins on the frustrated lattice hypothesized to become a quantum spin liquid that exhibit a spin glass transition in all these materials classes, not the "impurity" or witness spins.

Thus, herbertsmithite appears quite unique in that no spin glass transition occurs for the spins of the kagome planes and, instead, kagome-mediated witness spin interactions generate a spin glass of the "impurity" spins. This is highly distinct from the phenomena reported in refs. 56,63,75-86, and provides a unique new perspective on the $T \to 0$ ground state of herbertsmithite in which the kagome spins remain a candidate quantum spin liquid.

3. Spin noise in classical spin glasses

Magnetic noise has been used previously to study conventional spin glass compounds[87] and Josephson junction arrays[88,89], each of which generates strong magnetization signals. $1/f^{\alpha}$-type power spectral density is commonly observed in different classical spin glasses[87], while the quantitative characteristics vary among compounds. For example, in $Eu_{0.4}Sr_{0.6}S$, the spin noise power continues to grow below the glass transition[90]. Another example is $CdIn_{0.3}Cr_{1.7}S_4$ where the power exponent decreases above the glass transition at 16.6 K, but then starts increasing again above 17.5 K [91,92]. These observations differ from herbertsmithite's noise, naturally because the microscopic physics in these systems is unrelated to that of the witness spin interactions in a quantum spin liquid. Moreover, the far more technically advanced systems developed recently, including

achievement of sensitivity optimization reaching $fT/\sqrt{Hz}$ clean noise floor and millikelvin spin-noise measurements, have been introduced specifically to probe spin liquids[29,30,32].

68 Dommange, S., Mambrini, M., Normand, B. & Mila, F. Static impurities in the $S = 1/2$ kagome lattice: Dimer freezing and mutual repulsion. *Phys. Rev. B* **68**, 224416 (2003).

69 Gregor, K. & Motrunich, O. I. Nonmagnetic impurities in the spin-1/2 kagome antiferromagnet. *Phys. Rev. B* **77**, 184423 (2008).

70 Yang, J. & Li, T. Strong relevance of zinc impurities in spin-1/2 kagome quantum antiferromagnets: A variational study. *Phys. Rev. B* **109**, 115103 (2024).

71 Kimchi, I., Sheckelton, J. P., McQueen, T. M. & Lee, P. A. Scaling and data collapse from local moments in frustrated disordered quantum spin systems. *Nat. Commun.* **9**, 4367 (2018).

72 Kimchi, I., Nahum, A. & Senthil, T. Valence Bonds in Random Quantum Magnets: Theory and Application to $YbMgGaO_4$. *Phys. Rev. X* **8**, 031028 (2018).

73 Pal, S. *et al.* Gapped magnetic ground state in the spin-liquid candidate κ-(BEDT-TTF)$_2Ag_2(CN)_3$ suggested by magnetic spectroscopy. *Phys. Rev. B* **111**, L220404 (2025).

74 Aeppli, G., Shapiro, S. M., Maletta, H., Birgeneau, R. J. & Chen, H. S. Spin correlations near the ferromagnetic-to-spin-glass crossover (invited). *J. Appl. Phys.* **55**, 1628–1633 (1984).

75 Bert, F. *et al.* Ground State of the Kagomé-Like $S = 1/2$ Antiferromagnet Volborthite $Cu_3V_2O_7(OH)_2{\cdot}2H_2O$. *Phys. Rev. Lett.* **95**, 087203 (2005).

76 Li, Y. $YbMgGaO_4$: A Triangular-Lattice Quantum Spin Liquid Candidate. *Adv. Quantum Technol.* **2**, 1900089 (2019).

77 Ma, Z. *et al.* Spin-Glass Ground State in a Triangular-Lattice Compound $YbZnGaO_4$. *Phys. Rev. Lett.* **120**, 087201 (2018).

78 Shockley, A. C., Bert, F., Orain, J.-C., Okamoto, Y. & Mendels, P. Frozen State and Spin Liquid Physics in $Na_4Ir_3O_8$: An NMR Study. *Phys. Rev. Lett.* **115**, 047201 (2015).

79 Okamoto, Y., Nohara, M., Aruga-Katori, H. & Takagi, H. Spin-Liquid State in the *S* = 1/2 Hyperkagome Antiferromagnet $Na_4Ir_3O_8$. *Phys. Rev. Lett.* **99**, 137207 (2007).
80 Dally, R. *et al.* Short-Range Correlations in the Magnetic Ground State of $Na_4Ir_3O_8$. *Phys. Rev. Lett.* **113**, 247601 (2014).
81 Ramirez, A. P., Espinosa, G. P. & Cooper, A. S. Strong frustration and dilution-enhanced order in a quasi-2D spin glass. *Phys. Rev. Lett.* **64**, 2070–2073 (1990).
82 Bono, D., Limot, L., Mendels, P., Collin, G. & Blanchard, N. Correlations, spin dynamics, defects: the highly frustrated kagomé bilayer. *Low Temp. Phys.* **31**, 704–721 (2005).
83 Yang, J. *et al.* Spin jam induced by quantum fluctuations in a frustrated magnet. *Proc. Natl. Acad. Sci. U.S.A.* **112**, 11519–11523 (2015).
84 Wills, A. S. & Harrison, A. Structure and magnetism of hydronium jarosite, a model Kagomé antiferromagnet. *J. Chem. Soc., Faraday Trans.* **92**, 2161–2166 (1996).
85 Bisson, W. G. & Wills, A. S. Anisotropy-driven spin glass transition in the kagome antiferromagnet hydronium jarosite, $(H_3O)Fe_3(SO_4)_2(OH)_6$. *J. Phys.: Condens. Matter* **20**, 452204 (2008).
86 Petrenko, O. A., Ritter, C., Yethiraj, M. & McK Paul, D. Investigation of the Low-Temperature Spin-Liquid Behavior of the Frustrated Magnet Gadolinium Gallium Garnet. *Phys. Rev. Lett.* **80**, 4570–4573 (1998).
87 Weissman, M. B. $1/f$ noise and other slow, nonexponential kinetics in condensed matter. *Rev. Mod. Phys.* **60**, 537–571 (1988).
88 Koch, R. H. *et al*. Flicker ($1/f$) Noise in Tunnel Junction DC Squids. *J. Low Temp. Phys.* **51**, 207-224 (1983)
89 Candia, S., Leemann, C., Mouaziz, S. & Martinoli, P. Investigation of vortex dynamics in Josephson junction arrays with magnetic flux noise measurements. *Physica C: Supercond.* **369**, 309–312 (2002).
90 Reim, W., Koch, R. H., Malozemoff, A. P., Ketchen, M. B. & Maletta, H. Magnetic Equilibrium Noise in Spin-Glasses: $Eu_{0.4}Sr_{0.6}S$. *Phys. Rev. Lett.* **57**, 905–908 (1986).
91 Alba, M., Hammann, J., Ocio, M., Refregier, Ph. & Bouchiat, H. Spin-glass dynamics from magnetic noise, relaxation, and susceptibility measurements (invited). *J. Appl. Phys.* **61**, 3683–3688 (1987).

92 Ocio, M., Hammann, J., Refregier, Ph. & Vincent, E. Experimental investigation on the spin glass dynamics in $CdIn_{0.3}Cr_{1.7}S_4$ from noise measurements. *Physica B+C* **150**, 353–360 (1988).
